# Using ‘sentinel’ plants to improve early detection of invasive plant pathogens

**DOI:** 10.1371/journal.pcbi.1010884

**Published:** 2023-02-02

**Authors:** Francesca A. Lovell-Read, Stephen Parnell, Nik J. Cunniffe, Robin N. Thompson

**Affiliations:** 1 Mathematical Institute, University of Oxford, Oxford, United Kingdom; 2 Warwick Crop Centre, School of Life Sciences, University of Warwick, Coventry, United Kingdom; 3 Department of Plant Sciences, University of Cambridge, Cambridge, United Kingdom; 4 Mathematics Institute, University of Warwick, Coventry, United Kingdom; 5 Zeeman Institute for Systems Biology and Infectious Disease Epidemiology Research, University of Warwick, Coventry, United Kingdom; Stony Brook University, UNITED STATES

## Abstract

Infectious diseases of plants present an ongoing and increasing threat to international biosecurity, with wide-ranging implications. An important challenge in plant disease management is achieving early detection of invading pathogens, which requires effective surveillance through the implementation of appropriate monitoring programmes. However, when monitoring relies on visual inspection as a means of detection, surveillance is often hindered by a long incubation period (delay from infection to symptom onset) during which plants may be infectious but not displaying visible symptoms. ‘Sentinel’ plants–alternative susceptible host species that display visible symptoms of infection more rapidly–could be introduced to at-risk populations and included in monitoring programmes to act as early warning beacons for infection. However, while sentinel hosts exhibit faster disease progression and so allow pathogens to be detected earlier, this often comes at a cost: faster disease progression typically promotes earlier onward transmission. Here, we construct a computational model of pathogen transmission to explore this trade-off and investigate how including sentinel plants in monitoring programmes could facilitate earlier detection of invasive plant pathogens. Using *Xylella fastidiosa* infection in *Olea europaea* (European olive) as a current high profile case study, for which *Catharanthus roseus* (Madagascan periwinkle) is a candidate sentinel host, we apply a Bayesian optimisation algorithm to determine the optimal number of sentinel hosts to introduce for a given sampling effort, as well as the optimal division of limited surveillance resources between crop and sentinel plants. Our results demonstrate that including sentinel plants in monitoring programmes can reduce the expected prevalence of infection upon outbreak detection substantially, increasing the feasibility of local outbreak containment.

## Author summary

Plant diseases affect the environment and the economy negatively, with implications for biodiversity and food security. Fast detection of invading pathogens is essential to prevent widespread transmission. This is challenging, however, because many plant diseases have a long presymptomatic period (delay from initial infection to symptom onset) in globally important hosts. During this presymptomatic period, infection may spread undetected to other plants. While the presymptomatic period can be long, plant diseases often affect multiple host species, with different epidemiological characteristics. This provides an opportunity for planting sentinel plants, which are alternative host species that display visible symptoms of infection quickly, as early warning beacons for infection. In this research article, we use mathematical modelling to explore the potential for sentinel plants to aid plant disease monitoring programmes. We show that, for a high-profile plant pathogen (*Xylella fastidiosa*, which is currently devastating olive groves in southern Europe), the use of sentinel plants allows new outbreaks to be identified quickly, reducing the prevalence of infection when outbreaks are detected. Model simulations also indicate that our results apply more generally. Sentinel plants have the potential to assist fast detection of a wide range of invading plant pathogens.

## 1. Introduction

Infectious disease outbreaks in plant populations have devastating economic, environmental and societal consequences [1–8]. The global plant trade means that the spread of invasive plant pathogens poses an ever-increasing threat to international biosecurity [9,10]. Developing efficient and cost-effective methods for surveillance and control of invasive plant pathogens is therefore a vital area of current research [11–16].

Mathematical modelling is increasingly used to guide surveillance and intervention strategies for plant pathogens [11,14,17–19], helping policy-makers understand how to direct limited resources for control to reduce transmission [20–25]. Multiple studies of different pathogens have focused on the question of how to optimise control measures when a pathogen is known to be in a particular host landscape (‘reactive’ control). For example, using citrus canker (a bacterial disease of citrus plants) in Florida as a case study, Cunniffe *et al*. [26] showed how roguing (removal of confirmed infected plants) can be extended to removal of all plants in the proximity of a confirmed infected host. They demonstrated a modelling approach for identifying the optimal removal radius around confirmed infected hosts, balancing the reduction in the risk of further spread with the cost of destroying potentially healthy plants. Similarly, White *et al*. [27] investigated the efficacy of ‘buffer zones’ for intensive surveillance on the border of a region of known infection, and Adrakey *et al*. [28] developed a Bayesian approach for prioritising the removal of infected hosts based on the infection risk they pose to other plants.

While reactive control has been well-studied, a key additional challenge is designing surveillance methods specifically to achieve early outbreak detection [29]. This increases the chance of eradicating the pathogen from the landscape before it becomes widespread (‘preventive’ control). Parnell *et al*. [30,31] used probabilistic techniques to derive a simple ‘rule of thumb’ relating the expected prevalence of infection upon discovery to the sampling effort. This approach was extended by Mastin *et al*. [32] to a setting in which pathogen transmission via vectors is modelled explicitly, and used to investigate how to divide sampling resources optimally between hosts and vectors. Alonso-Chavez *et al*. [33] also applied this technique to explore the use of within-nursery surveillance for reducing the risk of growers selling infected plant material.

For many plant pathogens, a major obstacle to achieving early detection is a long incubation period (the time between initial infection and symptom onset; this has also been referred to as the cryptic, asymptomatic or presymptomatic period) [18,33–38]. If transmission by infected hosts can occur prior to symptom onset, monitoring programmes that rely upon visual inspection of potential hosts for signs of infection (as are standard across plant health [39,40]) may fail to identify the presence of a pathogen before widespread transmission has occurred. Indeed, Alonso-Chavez *et al*. [33] showed that if presymptomatic transmission occurs and the pathogen is discovered early in an outbreak, the expected prevalence of infection in the population at the time of discovery increases exponentially with the duration of the incubation period.

The incubation period of a pathogen may, however, vary substantially between different host species, or between hosts of different ages [35–37,41,42]. This suggests that in some cases there may be the potential to use alternative hosts with relatively short incubation periods as ‘sentinels’ to detect new outbreaks. In this context, we refer to a sentinel as a susceptible plant species specifically chosen to have a short incubation period, which is placed amongst the crop plants and regularly monitored for visible signs of infection (we note that, in the literature, the term ‘sentinel’ has alternatively been used to refer to plant species that are grown outside of their natural habitat and monitored to assess whether pests native to the new location pose a risk to that species, which is distinct from the context of this study [43]).

The rapid onset of visible symptoms in sentinel plants could result in earlier detection of the pathogen in the population. If this leads to a reduced prevalence of infection upon discovery, this could reduce the cost of reactive control. However, sentinels may also have drawbacks. The increased rate of symptom development in sentinel plants could result in more rapid onward transmission, which may counteract the positive effect of early detection and lead to an increased discovery prevalence. Therefore, research is needed to understand this trade-off and infer the conditions under which sentinel plants are likely to be beneficial for reducing the discovery prevalence. If sentinel plants are deployed, one key consideration is how many sentinel plants should be added to the population to provide sufficient opportunity for early detection whilst limiting the concurrent increase in the transmission rate. Another important question is how to divide limited surveillance resources optimally between crop and sentinel plants.

Although using sentinel plants to facilitate early detection of invasive pathogens has been suggested as a possibility previously [43,44], the question of how to design effective surveillance strategies using sentinel hosts targeted at a specific pathogen in a given region has not yet been addressed [43]. Here, we explore the potential for sentinel plants to aid early outbreak detection, using a plant pathogen of significant current importance as a case study (*Xylella fastidiosa*–see below). We construct a stochastic compartmental model of pathogen transmission that includes two different host types (crops and sentinels), and consider monitoring programmes in which, at regular intervals, a fixed number of plants are sampled at random and inspected for visible disease symptoms. A surveillance strategy is defined by: i) the number of sentinel plants added to the population; ii) the number of crops and sentinels to be examined in each sampling round, and; iii) the time interval between successive sampling rounds. For a given surveillance strategy, we use model simulations to calculate the expected detection prevalence (EDP) of the pathogen in the crop population at the time of discovery. We investigate the conditions under which including sentinel plants in a surveillance programme allows us to attain a lower EDP than standard monitoring (i.e., the analogous surveillance strategy but without any sentinel plants). For a given choice of sample size and sample interval, we apply a Bayesian optimisation algorithm to determine the minimum attainable EDP and the surveillance strategy for which this is achieved.

We demonstrate that including sentinel plants in a surveillance programme has the potential to reduce the EDP compared to a standard monitoring programme of equivalent sampling effort. Sentinel plants are particularly beneficial when limited resources are available for plant disease surveillance. We show that both the total number of sentinels deployed and the division of the sample between crop and sentinel plants are crucial in determining the effectiveness of a surveillance strategy. It can be preferable to sample a mixture of both sentinel and crop plants, rather than exclusively sampling sentinels. Overall, our results demonstrate that sentinel plants are a useful tool to improve early detection monitoring, and encourage further research to identify the range of host-pathogen systems for which sentinel plants can reduce the damage caused by invading plant pathogens.

### 1.1 Case study: *Xylella fastidiosa* infection in *Olea europaea* (European olive)

An important example of a pathogen for which the development of effective surveillance strategies is currently critical is *Xylella fastidiosa* [45], a vector-borne bacterial pathogen first isolated from infected grapevines in 1978 [46]. It is pathogenic to over 600 host plant species [47], including economically important crops such as olives, grapevines, almonds, citrus and coffee [48,49]. Depending on the host species and specific bacterial strain, symptoms of *X*. *fastidiosa* infection include leaf tissue necrosis (leaf scorch), stunted growth, decrease in fruit production, dieback and eventual death [36,48,49]. Outbreaks of *X*. *fastidiosa* in commercial crops therefore have substantial negative economic effects [22,50,51].

Of particular current concern is a strain of *X*. *fastidiosa*, subspecies *pauca*, known as CoDiRO (Complesso del Disseccamento Rapido dell’Olivo—loosely, ‘rapid drying disease of olive trees’), which was discovered in the Apulia region of south-east Italy in 2013 and subsequently identified as the causative agent of Olive Quick Decline Syndrome (OQDS) in that region [27,52]. CoDiRO spreads rapidly, is difficult to contain, and results in significant crop loss, threatening olive farming throughout Europe [22,35,48,53–55]. Recent projections indicate that the economic impact on olive farming in Italy, Greece and Spain alone could exceed €24 billion over the next 50 years if feasible control strategies are not devised [22]. However, the wide range of possible hosts for *X*. *fastidiosa* means that this pathogen poses a risk to European agriculture on an even broader scale [35,54–56].

Despite ongoing research, there is currently no effective treatment for OQDS, or *X*. *fastidiosa* infection more generally [48,49]. Control methods mainly consist of roguing infected plants and removing healthy plants in their vicinity, and reducing vector transmission using insecticides [27]. However, these interventions are costly, and must be swift in order to be effective [26,55,57]. The sooner control interventions are implemented, the less opportunity there is for the pathogen to be exported to new locations [14,26]. Devising appropriate monitoring programmes to facilitate early detection is therefore critical to the success of containment strategies [35].

*X*. *fastidiosa* is a prime example of a pathogen for which the incubation period can provide a major obstacle to achieving early detection. *X*. *fastidiosa* subsp. *pauca* has a long incubation period in European olive (*Olea europaea*) with a mean duration of around 15 months [35], and transmission by infected hosts can occur prior to symptom onset [36]. Since surveillance strategies for OQDS typically rely upon visual inspection of potential hosts as a first line of defence [40,48], presymptomatic transmission significantly limits the efficacy of current infection monitoring programmes (although molecular tests are able to detect *X*. *fastidiosa* infection before symptom onset [58,59], the costs of large-scale presymptomatic sampling are prohibitive [40]). Despite its long incubation period in *O. europaea*, there is substantial variation in the incubation period of *X*. *fastidiosa* across its large host range, depending on factors such as plant species and age, pathogen subspecies, and climatic conditions [35,36]. For this reason, the use of sentinel plants for surveillance of *X*. *fastidiosa* is a clear possibility, and has been identified as a key research area in the G20 Meetings of Agricultural Chief Scientists [36].

Here, we consider the candidate sentinel plant species *Catharanthus roseus*, a herbaceous flowering plant commonly known as Madagascan periwinkle. *C*. *roseus* is a known host of *X*. *fastidiosa* subsp. *pauca*, with a mean time from infection to symptom onset of around seven weeks (a factor of nine times shorter than *X*. *fastidiosa* in *O*. *europaea*) [35]. Although native to Madagascar, *C*. *roseus* has a wide geographical distribution and is already found across Italy and the rest of Europe, making it an ecologically suitable choice as a sentinel host [60]. Additionally, the small size of *C*. *roseus* plants allows them to be intercropped in olive groves.

Although here we use *X*. *fastidiosa* infection in *O*. *europaea* as an important case study, our modelling framework is intended to be general and extensible. It may also be applied to investigate the use of sentinel plants against other invasive pathogens for which presymptomatic infection hinders existing monitoring approaches.

## 2. Methods

### 2.1 Transmission model

**Fig 1:**
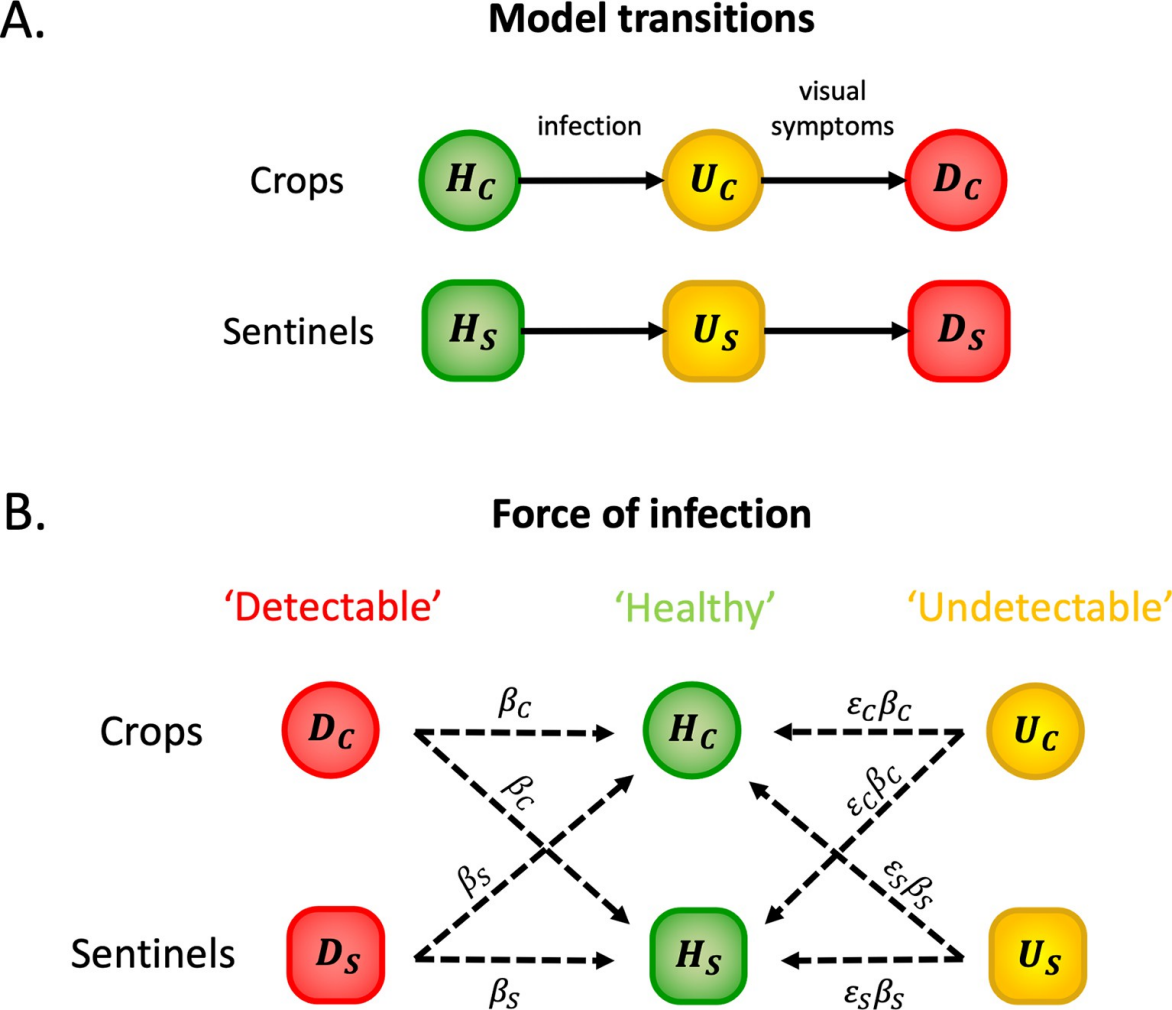
Schematic diagrams illustrating the compartments in the model (and how individual plants move between them) and the rates at which infections occur. A. Model transitions. For plants of either type (crop or sentinel), individual hosts begin in the Healthy compartment (*H*_*C*_ or *H*_*S*_) before moving to the corresponding Undetectable compartment (*U*_*C*_ or *U*_*S*_) upon infection. Undetectable plants progress to the appropriate Detectable compartment (*D*_*C*_ or *D*_*S*_) once visual symptoms develop. B. Force of infection. The rates at which different infectious hosts generate new infections. A Detectable crop plant can infect a Healthy crop or sentinel at rate *β*_*C*_ whilst an Undetectable crop can infect a Healthy crop or sentinel at the scaled rate *ε*_*C*_*β*_*C*_. Similarly, Detectable and Undetectable sentinels infect Healthy plants at rates *β*_*S*_ and *ε*_*S*_*β*_*S*_, respectively.

We considered a compartmental model of pathogen transmission in which plants are classified as ‘Healthy’ (*H*), ‘Undetectable’ (*U*) or ‘Detectable’ (*D*). The model includes two host types–crop plants (denoted by subscript *C*) and sentinel plants (denoted by subscript *S*). Healthy crops (*H*_*C*_) and sentinels (*H*_*S*_) are uninfected plants that are susceptible to infection. Undetectable crops (*U*_*C*_) and sentinels (*U*_*S*_) are plants that have been infected (and are infectious) but are not currently displaying visual symptoms. Once visual symptoms develop, plants progress into the Detectable compartment (*D*_*C*_ or *D*_*S*_ for crops and sentinels, respectively). A schematic illustrating the different compartments for both crop and sentinel plants is shown in Fig 1A.

We denote the total number of crop and sentinel plants in the population by *P*_*C*_ = *H*_*C*_+*U*_*C*_+*D*_*C*_ and *P*_*S*_ = *H*_*S*_+*U*_*S*_+*D*_*S*_, respectively, with a total population size of *P* = *P*_*C*_+*P*_*S*_. In each of our model simulations, fixed values of *P*, *P*_*C*_ and *P*_*S*_ are used, since we consider only the time until first detection and not the subsequent period during which infected plants and other nearby plants may be removed.

Undetectable and Detectable plants may generate new infections in any Healthy plant, with crops and sentinels assumed to be equally susceptible. The mode of transmission (insect vectors in the case of *X*. *fastidiosa*) is captured implicitly through the model parameterisation, rather than modelled explicitly. The parameters *β*_*C*_ and *β*_*S*_ represent the daily per capita rates at which individual infected Detectable crop and sentinel plants generate new infections, respectively. We also introduce the scaling parameters *ε*_*C*_ and *ε*_*S*_ to represent the relative infectiousness of Undetectable crops and sentinels compared to Detectable ones, so that the daily rates at which individual Undetectable crop and sentinel plants generate new infections are *ε*_*C*_*β*_*C*_ and *ε*_*S*_*β*_*S*_ respectively. In reality, Undetectable plants are unlikely to become infectious immediately after they are infected themselves. However, in the absence of detailed information on the time from infection to onset of infectiousness, we make a simplifying assumption that Undetectable plants are equally infectious throughout their presymptomatic period. To reflect the fact that Undetectable plants are likely to be less infectious than Detectable ones, we set 0<*ε*_*C*_, *ε*_*S*_<1. The different transmission routes and the corresponding rates at which infections occur are illustrated in Fig 1B. The mean duration of the crop and sentinel Undetectable (i.e. presymptomatic) periods are given by the parameters *γ*_*C*_ and *γ*_*S*_ respectively.

The resulting compartmental differential equation model representing pathogen transmission is given by

$$\frac{dH_{C}}{dt}=-\phi H_{C},\;\frac{dH_{S}}{dt}=-\phi H_{S},$$

$$\frac{dU_{C}}{dt}=\phi H_{C}-\frac{1}{\gamma_{C}}U_{C},\;\frac{dU_{S}}{dt}=\phi H_{S}-\frac{1}{\gamma_{S}}U_{S},$$ (1)

$$\frac{dD_{C}}{dt}=\frac{1}{\gamma_{C}}U_{C},\;\frac{dD_{S}}{dt}=\frac{1}{\gamma_{S}}U_{S},$$

where the force of infection, *ϕ*, acting on each Healthy plant is

$$\phi =\beta_{C}(\varepsilon_{C}U_{C}+D_{C})+\beta_{S}(\varepsilon_{S}U_{S}+D_{S}).$$ (2)

In our analyses, we run simulations of the analogous stochastic model using the direct method version of the Gillespie stochastic simulation algorithm, as described in Section 2.5.

### 2.2 The baseline case—Reduced model in the absence of sentinel plants

In the absence of sentinel plants (*P*_*S*_ = 0), the transmission model reduces to

$$\frac{dH_{C}}{dt}=-\phi_{b}H_{C},\;\frac{dU_{C}}{dt}=\phi_{b}H_{C}-\frac{1}{\gamma_{C}}U_{C},\;\frac{dD_{C}}{dt}=\frac{1}{\gamma_{C}}U_{C},$$ (3)

where the force of infection, *ϕ*_*b*_, acting on each Healthy plant is

$$\phi_{b}=\beta_{C}(\varepsilon_{C}U_{C}+D_{C}).$$ (4)

Throughout, this reduced system is what we will refer to as the ‘baseline case’ for a particular choice of model parameterisation or surveillance strategy. It provides a point of reference for the EDP, allowing us to determine whether the EDP is reduced or increased when sentinel plants are introduced. Specifically, the effect of including sentinel plants in a surveillance strategy may then be assessed relative to the baseline case with equivalent parameterisation and sampling effort (i.e. the same total sample size and sample interval). When considering the baseline case, we again run model simulations of the analogous stochastic model using the direct method version of the Gillespie stochastic simulation algorithm.

### 2.3 Sampling and detection

We considered a monitoring programme in which different random samples of *N* hosts are taken from the population every Δ days and inspected for symptoms of disease. We chose random sampling since, in a “well-mixed” model that assumes spatial homogeneity in the infection risk across the landscape, it outperforms repeated sampling of the same hosts (see S1 Text and S1 Fig), whilst being straightforward to implement computationally. For a given surveillance strategy (choice of *N* and Δ), we fix the number of crop plants and sentinel plants in the sample so that *N* = *N*_*C*_+*N*_*S*_, where *N*_*C*_ is the crop sample size and *N*_*S*_ is the sentinel sample size (in the baseline case, *N*_*S*_ = 0 and *N*_*C*_ = *N*). To reflect the introduction of the pathogen at a random time relative to the sampling scheme, we begin sampling from our model disease system at a time selected uniformly at random from the interval [0, Δ]. We then sample every Δ days until detection occurs. In our analyses, we assume that Detectable plants in a sample are always correctly identified as being infected, Undetectable plants are never correctly identified as being infected, and Healthy plants are always correctly identified as uninfected. Therefore, detection occurs at a given time if and only if at least one Detectable plant (crop or sentinel) is included in the sample selected at that time.

### 2.4 Model parameterisation

We selected the epidemiological parameters of our model (Table 1) to be consistent with *X*. *fastidiosa* subsp. *pauca* infection in the crop plant *O*. *europaea* (European olive) and the sentinel plant *C*. *roseus* (Madagascan periwinkle) [35,61]. In our main analyses, we assumed that the transmission coefficient for Detectable sentinels (*β*_*S*_) was equal to that for Detectable crops (*β*_*C*_). In other words, infected sentinel plants were assumed to generate the same number of infections (on average) as infected crop plants. Whilst some studies have suggested that *C*. *roseus* may be more susceptible to *X*. *fastidiosa* infection than *O*. *europaea* [62], others have indicated that the rate at which the vector acquires the pathogen from *C*. *roseus* is lower than from *O*. *europaea* [63], and detailed information on how these conflicting factors affect the overall transmission rates is lacking. We were also required to assume the relative infectiousness of Undetectable sentinels compared to Detectable sentinels. The value chosen (*ε*_*S*_ = 0.1) is a conservative choice compared to the equivalent parameter for crop plants (*ε*_*C*_ = 0.015) since it assumes that Undetectable sentinels are substantially more infectious than Undetectable crops. This choice aims to provide a lower bound on the utility of sentinel plants. Due to the uncertainty in these parameter values, we performed extensive sensitivity analyses to determine how the choice of parameterisation affected our results (Section 3.4, S2 Text, S1–S2 Tables and S2–S10 Figs). In each case that we considered, our overall finding–that sentinel hosts can be helpful to reduce the EDP–was unchanged.

**Table 1:** The epidemiological parameters of the model, their meanings, and their baseline values chosen to be consistent with *X*. *fastidiosa* infection in *O*. *europaea* (crop) and *C*. *roseus* (sentinel). Other model parameter values are considered in S2 Text, S1–S2 Tables, and S2–S10 Figs.

Parameter	Meaning	Value	Justification
*β*_*C*_	Transmission coefficient for Detectable crops	5×10^−5^	Chosen so that *β*_*C*_*P*_*C*_ = 0.05 [61]
*β*_*S*_	Transmission coefficient for Detectable sentinels	5×10^−5^	Assumed
*ε*_*C*_	Transmission scaling factor for Undetectable crops	0.015	[61]
*ε*_*S*_	Transmission scaling factor for Undetectable sentinels	0.1	Assumed
*γ*_*C*_	Mean duration of crop Undetectable period	452 days	[35]
*γ*_*S*_	Mean duration of sentinel Undetectable period	49 days	[35]

Throughout, we considered a crop population of *P*_*C*_ = 1000 plants. The size of the sentinel population, *P*_*S*_, varied between simulations. We initialised each simulation with a single Undetectable infected host, with the probability of that host being a crop or a sentinel plant determined by their respective proportions within the population.

### 2.5 Computational implementation

As noted above, for a given number of sentinels, *P*_*S*_, added to the population, we performed simulations of pathogen spread using the Gillespie stochastic simulation algorithm (direct method) [64], generating stochastic epidemic curves in which the numbers of Undetectable and Detectable crops and sentinels were tracked over time until the entire population became infected (S11A Fig). Then, given the remaining parameters defining the surveillance strategy (values of *N*, *N*_*C*_, *N*_*S*_ and Δ), we implemented the corresponding monitoring programme on these simulated epidemics as described in Section 2.3 (S11B and S11C Fig). For each sampling run and subsequent detection completed on a unique epidemic curve, we recorded the total prevalence of infection in crop plants when the pathogen was discovered (i.e., we recorded the value of *U*_*C*_+*D*_*C*_ on discovery) (S11C Fig). Repeatedly implementing the same surveillance strategy on many simulated epidemic curves, we obtained a distribution on the discovery prevalence amongst crop plants for that surveillance strategy and computed the EDP as the mean value of that distribution (note that this does not include the prevalence amongst sentinel plants, since we assume that damage to the crop population is the primary concern for commercial growers) (S11D Fig). For any given choice of *N* and Δ, the baseline EDP for that strategy is computed in the same way, setting *P*_*S*_ (and thus also *N*_*S*_) equal to 0. Throughout, the EDP for a given surveillance strategy (choice of *P*_*S*_, *N*_*C*_, *N*_*S*_, and Δ) was calculated by performing sampling on 25,000 simulated epidemic curves.

**Fig 2:**
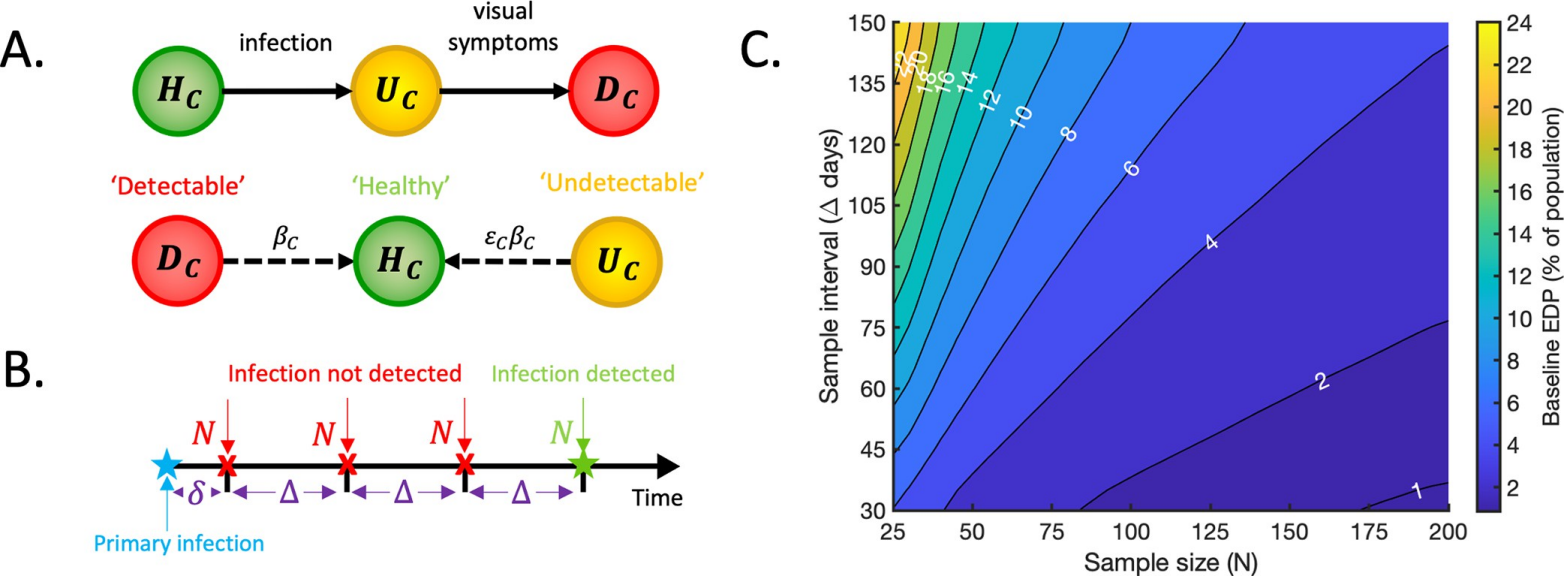
The baseline case–the model in the absence of sentinel plants. A. Schematic illustrating how crop plants progress through the model compartments, and the rates at which transmissions occur. Individual hosts begin in the Healthy compartment (*H*_*C*_), move to the Undetectable compartment (*U*_*C*_) upon infection and progress to the Detectable compartment (*D*_*C*_) once visual symptoms develop. A Detectable crop infects Healthy crops at per capita rate *β*_*C*_ whilst an Undetectable crop generates infections at the scaled per capita rate *ε*_*C*_*β*_*C*_. B. Schematic illustrating the implementation of the monitoring programme. Monitoring begins at a random time *δ* relative to the time of primary infection, where *δ* is drawn from a *U*[0, Δ] distribution. Random samples of size *N* are subsequently selected from the population at regular time intervals Δ. Infection is detected at a given time if a Detectable plant is contained in the sample selected at that time. C. The baseline EDP, expressed as a percentage of the total crop population size, as the sample size (*N* = *N*_*C*_) and sample interval (Δ) vary.

Initially, we considered the effects of implementing a monitoring programme without sentinel plants (the baseline case described in Section 2.2) (Section 3.1). A schematic of the reduced version of the model system in this case is shown in Fig 2A. In the absence of sentinel plants, the monitoring programme requires selecting a random sample of *N*_*C*_ = *N* plants at regular time intervals Δ and checking for the presence of Detectable plants (*D*_*C*_) in the sample (Fig 2B). Doing so, we computed the baseline EDP for sample sizes *N*_*C*_ = 25, 30, 35, …, 200 and sample intervals Δ = 30, 35, 40, …, 150 days (Fig 2C).

Next, we considered three fixed values for the number of sentinels (*P*_*S*_) added to the population: *P*_*S*_ = 50, *P*_*S*_ = 100 and *P*_*S*_ = 200 (Section 3.2). We allowed the total sample size *N* to take values *N* = 25, 50, 75, …, 200, and considered five values for the sample interval: Δ = 30, 60, 90, 120 and 150 days. In each case, we allowed the number of sentinels included in the sample (*N*_*S*_) to be chosen in the range [0, min(*P*_*S*_, *N*)], and applied a Bayesian optimisation algorithm ([65,66]; see also S3 Text) to identify the choice of *N*_*S*_ for which the EDP was maximally reduced compared to the baseline level. The choice of upper limit on *N*_*S*_ ensures that the number of sentinels sampled does not exceed the total sample size (*N*) or the total number of sentinels available (*P*_*S*_).

Finally, we considered varying the number of sentinels in the population (*P*_*S*_) and in the sample (*N*_*S*_) simultaneously and sought to identify the pair of values $\left(P_{S}^{*},N_{S}^{*}\right)$ that maximised the reduction in EDP compared to the baseline (Section 3.3). In that case, we allowed the total sample size *N* to take values *N* = 25, 30, 35, …, 200, and the sample interval to take values Δ = 30, 35, 40, …, 150 days. For each pair of (*N*, Δ) values we allowed the total number of sentinels added to the population (*P*_*S*_) to vary in the range [0,350], and the number of sentinels included in the sample (*N*_*S*_) to vary in the range [0, min(*P*_*S*_, N)]. The upper bound of 350 on *P*_*S*_ was selected following trial simulations that indicated this would be sufficient to identify the optimal value of *P*_*S*_ across the (*N*, Δ) range considered. For each (*N*, Δ) pair, we applied a Bayesian optimisation algorithm to determine the optimal values of *P*_*S*_ and *N*_*S*_, and constrained it to ensure that the number of sentinels included in the sample could not exceed the total number of sentinels available in the population (S3 Text).

## 3. Results

### 3.1 The baseline case–a monitoring programme without sentinel plants

We first considered the effects of implementing a monitoring programme without sentinel plants (the baseline case described in Section 2.2). As expected, lower EDPs were achieved with larger sample sizes *N*_*C*_ (inspecting more plants) and smaller sample intervals Δ (inspecting more frequently) (Fig 2C). These baseline values provide a point of comparison that we will use to evaluate the relative effects of sentinel-based strategies in subsequent sections. We also performed sensitivity analyses for different baseline parameter values (S2 Text, S1 Table and S2 and S3 Figs). In every case that we considered, the qualitative behaviour of the baseline EDP as *N* and Δ were varied was unchanged.

### 3.2 Introducing sentinel plants–choosing *P*_*S*_ and *N*_*S*_ carefully is critical

We next considered introducing sentinel plants to the population using the full model described in Section 2.1. This raises two important questions.

- **How many sentinels should we add to the population (*P*_*S*_)?** Although the relatively fast symptom development of sentinels facilitates the rapid detection of disease, this is only beneficial if the faster discovery time corresponds to a lower EDP. Since adding sentinels will also increase the rate of pathogen transmission, including too many sentinels negates the benefits of fast detection, particularly if (as assumed here) sentinel plants are more infectious than crop plants when Undetectable.
- **How many of those sentinels should we include in the sample (*N*_*S*_)?** Although a natural choice may be to sample preferentially from the available sentinel population (i.e. to include as many sentinels as possible in the sample), this is not necessarily optimal. For example, if the number of sentinels in the population is close to the sample size, this strategy would lead to frequent repeated sampling of the same set of plants, resulting in a reduction in the information gained per sample (see S1 Text and S1 Fig).

**Fig 3:**
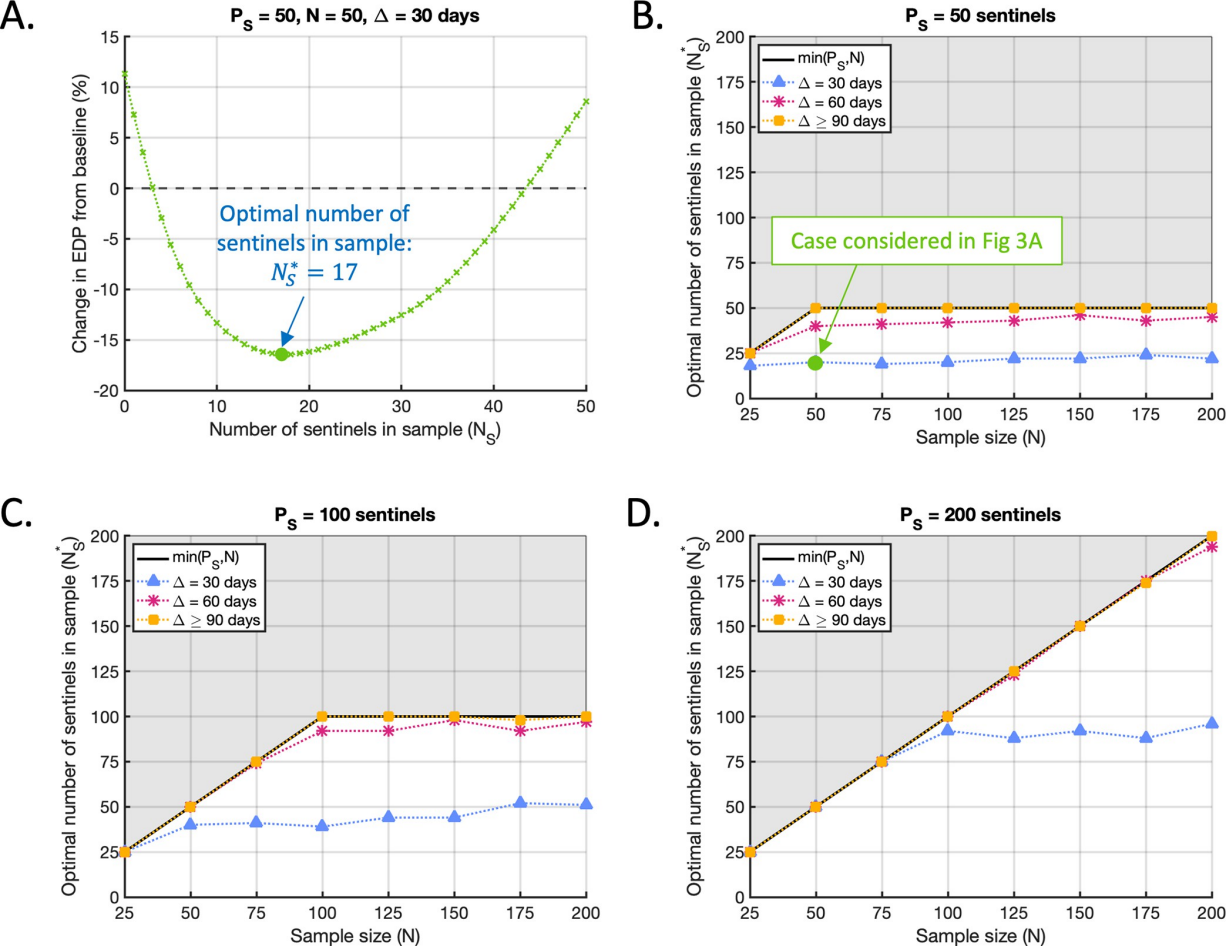
The optimal number of sentinel plants to include in the sample depends on the sample size, sample interval and the total number of sentinels in the population. A. The effect of varying the number of sentinels included in the sample (*N*_*S*_) on the percentage change in EDP compared to the baseline level, in the case *P*_*S*_ = 50, *N* = 50, Δ = 30 days. The number of sentinels in the sample for which the reduction in EDP is maximised ($N_{S}^{*}=17$) is indicated by the green circle. Black dashed line marks the baseline EDP. B. The optimal number of sentinels $N_{S}^{*}$ to include in the sample when *P*_*S*_ = 50, as the sample size (*N*) and sample interval (Δ) vary. Solid black line marks the maximum possible number of sentinels that could be sampled at any time (min(*P*_*S*_, *N*)). Grey shading marks the unfeasible region in which *N*_*S*_ exceeds this maximum. Green circle marks the case considered in A (*P*_*S*_ = 50, *N* = 50, Δ = 30 days). C. The analogous figure to B, but with *P*_*S*_ = 100 sentinels added to the population. D. The analogous figure to B, but with *P*_*S*_ = 200 sentinels added to the population.

**Fig 4:**
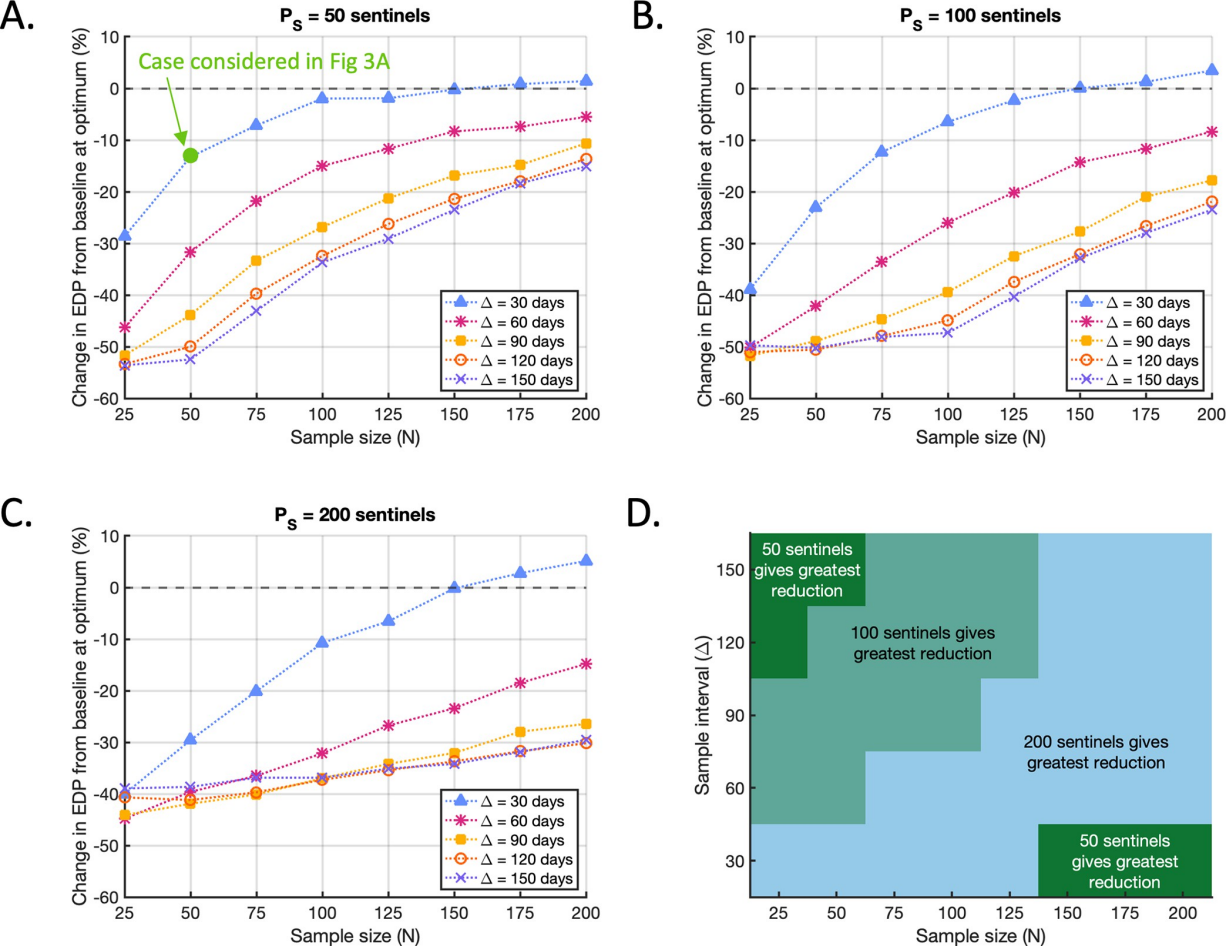
Optimal reductions in EDP compared to the baseline level. A. The best achievable percentage changes in the EDP compared to the baseline level for each (*N*, Δ) pair when *P*_*S*_ = 50, corresponding to the optimal strategies identified in Fig 3B. Green circle marks the case considered in Fig 3A (*P*_*S*_ = 50, *N* = 50, Δ = 30 days). Note that the baseline level depends on *N* and Δ (Fig 2C, S12A Fig), so the relative changes in EDP shown here are not a measure of the resultant EDP. The resultant EDP decreases with sampling effort (S12B, S12C and S12D Fig). B. The analogous figure to A, but with *P*_*S*_ = 100 sentinels added to the population and results corresponding to the strategies identified in Fig 3C. C. The analogous figure to A, but with *P*_*S*_ = 200 sentinels added to the population and results corresponding to the strategies identified in Fig 3D. D. Combinations of the sample size *N* and sample interval Δ for which adding *P*_*S*_ = 50 (dark green), *P*_*S*_ = 100 (light green) or *P*_*S*_ = 200 (blue) sentinels to the population led to the greatest reduction in the EDP compared to the baseline level (of the three values of *P*_*S*_ considered).

In this section, we demonstrate how choosing *P*_*S*_ and *N*_*S*_ carefully is critical to avoid the introduction of sentinel plants having a negative effect and instead achieve the maximum possible reduction in EDP for a given sampling effort. For almost all values of *P*_*S*_, *N* and Δ that we considered, when the number of sentinels included in the sample was optimised (as indicated in Fig 3), a reduction in the EDP compared to the baseline value was achieved (Figs 4A, 4B and 4C). However, when the number of sentinels in the population or the sample was chosen non-optimally, sentinel plants were less beneficial and in some cases detrimental (Fig 3A).

As described in Section 2.5, we considered three fixed values for the number of sentinels added to the population (*P*_*S*_ = 50, *P*_*S*_ = 100 and *P*_*S*_ = 200) and a range of sample sizes (*N*) and sample intervals (Δ). For each combination of (*P*_*S*_, *N*, Δ) that we considered, we ran the Bayesian optimisation algorithm (see Section 2.5 and S3 Text) to identify the choice of *N*_*S*_ corresponding to the greatest reduction in EDP compared to the baseline value for that (*N*, Δ) pair. We denoted this optimal choice of *N*_*S*_ by $N_{S}^{*}$. For example, in the case *P*_*S*_ = 50, *N* = 50, and Δ = 30 days, the optimisation indicated that the maximum reduction in EDP was achieved when $N_{S}^{*}=17$ sentinels were included in each sampling round (out of a total possible maximum of 50) (Fig 3A). This choice of sampling strategy (indicated by the green circle) led to a 16% reduction in the EDP compared to the baseline value. When *N*_*S*_ was instead chosen to take another of the values considered, smaller reductions (or even increases) in the EDP were achieved. The optimal number of sentinels $N_{S}^{*}$ to include in the sample across the range of sample sizes (*N*) and sample intervals (Δ) is shown for *P*_*S*_ = 50, 100 and 200 in Figs 3B, 3C and 3D respectively, with the corresponding reductions in the EDP compared to the baseline shown in Figs 4A, 4B and 4C.

The optimal number of sentinel plants to include in the sample depended strongly on the sample interval and on the relationship between the sample size and the total number of sentinels available (Figs 3B, 3C and 3D). When Δ = 90 days, 120 days or 150 days, the optimal strategy in every case we considered was to sample the maximum possible number of sentinels (i.e. $N_{S}^{*}=\min(P_{S},N)$). Since this result is identical for all three of those cases, they are represented by the single yellow line in Figs 3B, 3C and 3D. However, for the shorter sample intervals of Δ = 30 and 60 days (blue and pink lines respectively), the optimal monitoring strategy involved sampling a combination of sentinel plants and crop plants. In other words, in those scenarios it was preferable to sample fewer than the maximum allowable number of sentinels (i.e. $N_{S}^{*}<\min(P_{S},N)$) for a range of choices of *P*_*S*_ and *N*, particularly when the total number of sentinels in the population was not substantially larger than the sample size.

These results may be explained by noting that, if the total number of sentinels available to sample from (*P*_*S*_) is not substantially larger than the sample size (*N*), then sampling the maximum allowable number of sentinels (min(*P*_*S*_, *N*)) results in many or all of the same plants being repeatedly selected in every sampling round. If the sample interval is short, this leads to the frequent re-inspection of plants whose disease-free status was already established in the recent past, limiting the information gained per sampling round. However, this effect diminishes as the sample interval increases, because the disease status of plants inspected in the previous sample becomes less informative of their state at the next sample time. Thus, when the sample interval is large, sampling the maximum possible number of sentinels is the optimal strategy ($N_{S}^{*}=\min(P_{S},N)$) regardless of the sample size (*N*) or the total number of sentinels available (*P*_*S*_). These results confirm the need to consider the division of the sample between crops and sentinels as a variable quantity that should be chosen carefully based on the precise conditions under which surveillance is taking place. If the number of sentinels included in the sample is suboptimal, smaller reductions in the EDP will be achieved, and sentinel plants may even have a detrimental effect (Fig 3A).

As expected, the resultant EDP following the implementation of the optimal sentinel strategy decreased with greater sampling effort: taking larger samples and/or sampling more frequently always led to a lower EDP (S12 Fig). However, larger percentage reductions in the EDP relative to the baseline level were mostly achieved when the sampling effort was low (i.e. when the sample size was small and/or the sample interval was large) (Figs 4A, 4B and 4C). This is because, when the sampling effort was low, the baseline EDP was much higher to begin with (Fig 2C). In those cases, the potential for the use of sentinel plants to lead to a large relative improvement in the EDP was greater than when the sampling effort was high and the baseline EDP was already low.

As well as affecting the magnitude of the reduction in EDP compared to the baseline, the choice of sample size and sample interval also affected the total number of sentinels for which the greatest reduction was achieved (Fig 4D). For example, when the sample size was *N* = 25 and the sample interval was Δ = 150 days, choosing *P*_*S*_ = 50 led to the greatest reduction in EDP of the three values considered (54%, compared to a 50% reduction when *P*_*S*_ = 100 and a 39% reduction when *P*_*S*_ = 200). However, for *N* = 100 and Δ = 30 days, choosing *P*_*S*_ = 200 gave the greatest reduction in EDP (11%, compared to 2% and 6% when *P*_*S*_ = 50 and 100, respectively). Overall, introducing fewer sentinels was preferable when the sampling effort was either low or very high, with larger numbers preferable for intermediate sampling efforts (Fig 4D).

This variation in the optimal number of sentinels for different values of (*N*, Δ) reflects the crucial trade-off between the benefits and drawbacks of sentinel plants. Although adding sentinels to the population helps to facilitate early detection, it also leads to an increased rate of pathogen transmission (particularly if sentinels are more infectious than crop plants when Undetectable, as assumed here). Therefore, including more sentinel plants is only beneficial if the advantage gained from sampling them outweighs the impact of increased transmission.

For small sample sizes *N*, the capacity to exploit large numbers of sentinel plants is limited. Although increasing the number of sentinels is beneficial up to a point, since it allows for sampling without frequently inspecting the same sentinel plants, there is a threshold number of sentinels to introduce beyond which there will be no further improvement in detection to counterbalance the concurrent increase in overall transmission. The benefit of increasing *P*_*S*_ is more limited when the sample interval Δ is large, since in that scenario a past negative sample is less likely to indicate that the current sample will be negative. Thus, the same sentinels may be resampled without a substantial correlation between successive samples. Smaller numbers of sentinels are therefore preferable when the sampling effort is low (Fig 4D). At the opposite extreme, when sampling is very intensive (large sample size *N* and small sample interval Δ) then the baseline EDP is low (Fig 2C) and the potential for sentinel plants to reduce it is limited. In such a case, including a very large number of sentinels in the population is also not optimal, since this limited reduction is outweighed by the consequent higher rate of transmission. Therefore, smaller numbers of sentinels are also preferable when the sampling effort is very high (Fig 4D). However, for intermediate sampling efforts, larger numbers of sentinels perform better, since the capacity to exploit them and the scope to reduce the EDP compared to the baseline are less restricted. These results emphasise that judicious selection of the total number of sentinel plants added to the population is required to ensure that the benefits of including them are sufficient to offset their drawbacks in terms of increasing transmission. This emphasises the need for an epidemiological modelling framework as provided here to guide the number of sentinel plants to introduce, and we explore how the number of sentinel plants can be optimised in the next section.

### 3.3 Optimising the number of sentinel plants included in the population

We next considered optimising the total number of sentinel plants added to the population (*P*_*S*_) and the number of sentinels included in the sample (*N*_*S*_) simultaneously. As outlined in Section 2.5, for each (*N*, Δ) pair considered we applied a constrained Bayesian optimisation algorithm to identify the values $\left(P_{S}^{*},N_{S}^{*}\right)$ that reduced the EDP most compared to the baseline value.

**Fig 5:**
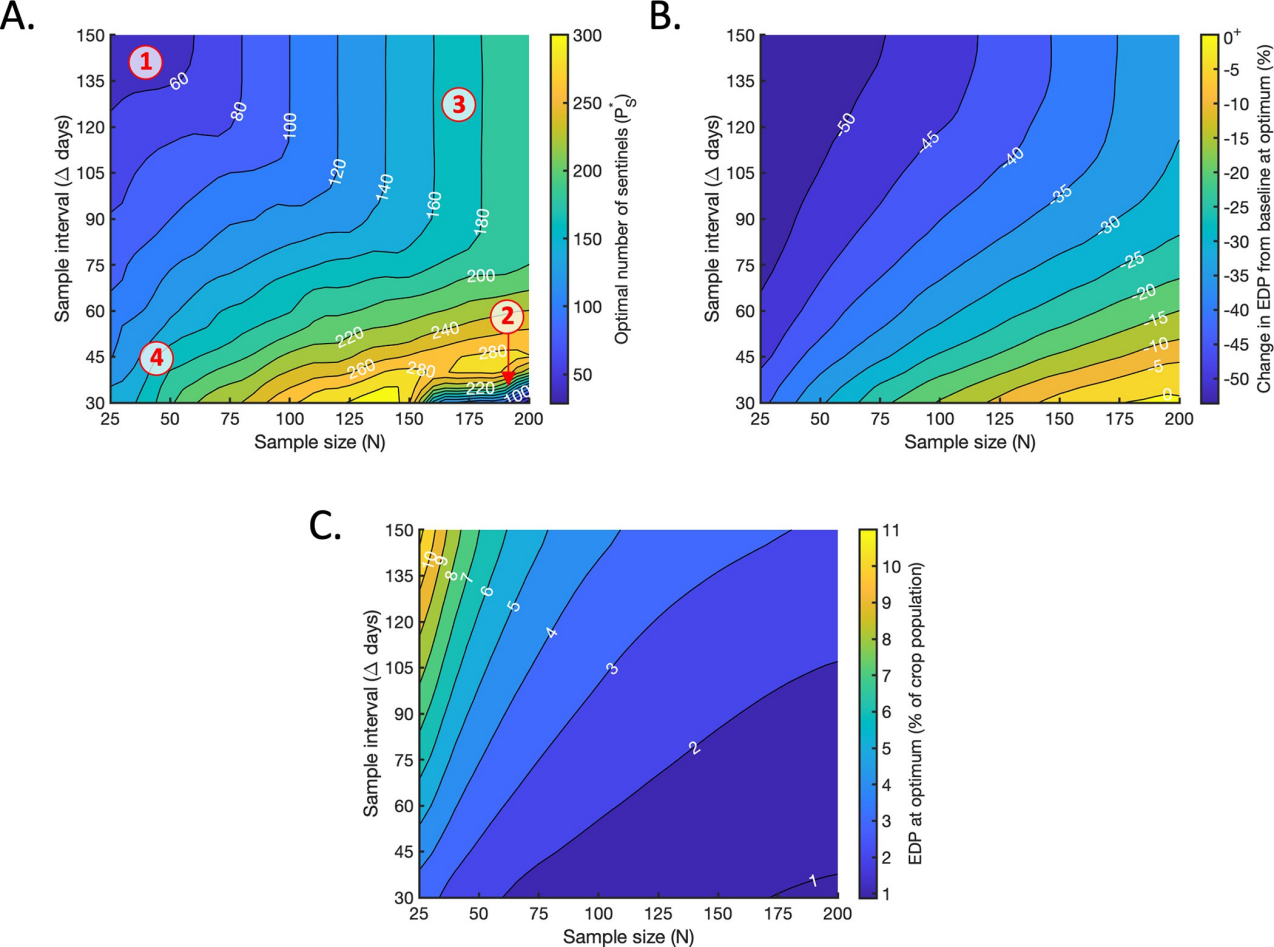
Optimising the number of sentinels to include in the population. A. The optimal number of sentinel plants to include in the population, $P_{S}^{*}$, for which the maximal reduction in the EDP compared to the baseline level is achieved (if *N*_*S*_ is also chosen optimally). Region 1: When the sampling effort was small, $P_{S}^{*}$ was low. $P_{S}^{*}$ increased for larger sample sizes (moving to the right on the figure) and smaller sample intervals (moving downwards on the figure). Region 2: $P_{S}^{*}$ dropped again when the sampling effort was very high. In that region, the baseline EDP was very low (Fig 2C), and the scope for reducing it insufficient to offset the increase in transmission rate caused by adding large numbers of sentinel plants into the population. Region 3: When the sample interval (Δ) was large, the optimal number of sentinel plants to include in the population was equal to the sample size (*N*) (contour lines are vertical). In that region, the sample interval was long enough to allow for repeated sampling of the same plants, eliminating the need for *P*_*S*_ to exceed the sample size. Region 4: When the sample interval (Δ) was small, the optimal total number of sentinels in the population was substantially larger than the sample size. In that region, a large sentinel population was necessary to avoid frequent repeated sampling of the same plants. B. The percentage change in the EDP compared to the baseline value at the optimum, achieved when $P_{S}=P_{S}^{*}$ and $N_{S}=N_{S}^{*}$. C. The resultant value of the EDP at the optimum, expressed as a percentage of the total crop population.

Including sentinel plants in the population was beneficial across the range of sampling strategies considered, with the optimal number of sentinels added to the population ($P_{S}^{*}$) greater than zero for all values of (*N*, Δ) (Fig 5A). This shows that deploying sentinel plants has the potential to reduce the EDP. However, the optimal number of sentinels to use varied substantially with the sample size (*N*) and sample interval (Δ). Consistent with Fig 4D, $P_{S}^{*}$ was low when the sampling effort was small (region 1 in Fig 5A), with the optimal number of sentinels increasing for larger sample sizes and smaller sample intervals. This is again due to the benefit of avoiding repeated sampling of the same plants when the sampling effort is high. By including more sentinels, the chance of repeatedly sampling the same sentinel plants is reduced. As in Fig 4D, $P_{S}^{*}$ dropped again when the sampling effort was very high (region 2 in Fig 5A). In that region, the baseline EDP was very low (Fig 2C), and the scope for reducing it insufficient to offset the increase in transmission rate caused by adding large numbers of sentinel plants into the population. When the sample interval (Δ) was large, the optimal number of sentinel plants to include in the population was equal to the sample size (*N*) (region 3 in Fig 5A in which the contour lines are vertical). In that region, the sample interval was long enough to allow for repeated sampling of the same plants, eliminating the need for *P*_*S*_ to exceed the sample size.

For almost all of the (*N*, Δ) values considered, the optimal number of sentinels to include in the sample ($N_{S}^{*}$) was the maximum possible (i.e. $N_{S}^{*}=min(P_{S},N)$) (S13 Fig). As observed in Section 3.2, preferential sampling of sentinel plants was always optimal when the sample interval (Δ) was large, since the same plants could be sampled repeatedly while still gaining new information about pathogen transmission each time. Sampling the maximum possible number of sentinels was also optimal when the sample interval and sample size were both small. In that region, the optimal total number of sentinels in the population was substantially larger than the sample size (region 4 in Fig 5A), meaning that preferential sampling of sentinels did not result in repeated sampling of the same plants. $N_{S}^{*}$ only fell below min(P_S_, N) when the sampling effort was very high (large *N* and small Δ). In that region, the total number of available sentinels dropped below the sample size (region 2 in Fig 5A) and the issue of repeated sampling again became relevant. However, since the sample size substantially exceeded the number of sentinels in that region, repeated sampling had a relatively small effect and the consequent reduction in the optimal proportion of sentinels to sample was not large (S13 Fig).

When *P*_*S*_ and *N*_*S*_ were chosen optimally, reductions in the EDP compared to the baseline value were achieved for almost all (*N*, Δ) values considered (Fig 5B). Of course, since the baseline itself (Fig 2C) can theoretically always be achieved by choosing *P*_*S*_ = 0, we would not expect the optimal resultant EDP to exceed the baseline substantially for any choice of *N* and Δ. However, in cases where the optimal sentinel strategy has little effect on the EDP, small increases in the EDP compared to the baseline may still occur due to the stochasticity of our simulations. This was observed for some very large values of *N* and very small values of Δ (Fig 5B).

As in all previous cases, the resultant EDP decreased with greater sampling effort: increasing the sample size (*N*) and/or decreasing the sample interval (Δ) always led to a smaller EDP (Fig 5C).

### 3.4 Robustness of the results to the parameter values used

As far as possible, the epidemiological parameters used in our analyses were chosen based on literature estimates for *X*. *fastidiosa* infection in *O*. *europaea* and *C*. *roseus* (Table 1). However, reported estimates were not available for all parameters. In particular, we were required to assume values for the transmission coefficient for Detectable sentinels (*β*_*S*_) and the transmission scaling factor for Undetectable sentinels (*ε*_*S*_) (see Section 2.4). Therefore, we also conducted supplementary analyses to determine how variation in the model parameters affected our results. These analyses are crucial for demonstrating the generalisability of our results. As well as varying *β*_*S*_ and *ε*_*S*_, we considered variation in the transmission scaling factor for Undetectable crops (*ε*_*C*_), the mean durations of the crop and sentinel Undetectable periods (*γ*_*C*_ and *γ*_*S*_), the crop population size (*P*_*C*_) and the initial number of Undetectable infected individuals (*U*_0_). For each of these, we generated plots analogous to Fig 5 for two different values of the relevant parameter (S2 Text, S1–S2 Tables and S4–S10 Figs).

Although the optimal total number of sentinel plants to include in the population ($P_{S}^{*}$; panels A and B in each of S4–S10 Figs) exhibited some variation with the changes in parameter values, the overall results remained qualitatively similar in most respects. In half of the cases that we considered, the main qualitative change was that the drop in $P_{S}^{*}$ observed in region 2 of Fig 5A did not occur (S4, S5, S8, S9 and S10 Figs). In general, this arose in cases in which sentinel plants became relatively more beneficial due to the parameter change–for example, when the mean duration of the sentinel Undetectable period (*γ*_*S*_) or the relative infectiousness of Undetectable sentinels (*ε*_*S*_) was reduced. In some other cases, when the parameter change led to sentinel plants becoming less beneficial, such as increasing the transmission coefficient for Detectable sentinels (*β*_*S*_) or the relative infectiousness of Undetectable sentinels (*ε*_*S*_), then smaller numbers of sentinels were preferable (S4, S6, S7 and S8 Figs). The optimal number of sentinels was also predictably reduced when the crop population size was halved (*P*_*C*_ = 500; S9 Fig), confirming that sentinel-based strategies must be assessed in context and tailored to the specific crop population being considered.

As expected, larger percentage reductions in the EDP compared to the baseline were observed for parameter changes that increased transmission amongst crop plants or that made sentinel plants relatively more beneficial (panels C and D in S4, S5, S6, S8 and S10 Figs). This included increasing the relative infectiousness of Undetectable crops, increasing the initial number of infected hosts, and decreasing the infectiousness of sentinels or the mean duration of their Undetectable period. Similarly, for parameter changes that made sentinel plants relatively less beneficial, such as increasing the mean duration of their Undetectable period or their relative infectiousness, smaller reductions in the EDP were observed (S4, S6 and S8 Figs).

The resultant EDP at the optimum (panels E and F in each figure) remained qualitatively similar with variation in each parameter. In every case, increasing the sampling effort (increasing the sample size *N* and/or reducing the sample interval Δ) led to a smaller resultant EDP. The resultant EDP also remained quantitatively similar for almost all parameter changes, with the only substantial change occurring when the transmission scaling factor for Undetectable crops was increased from *ε*_*C*_ = 0.015 to *ε*_*C*_ = 0.25 (S5 Fig).

## 4. Discussion

An important challenge in plant disease management is to detect invading pathogens before they become widespread in the host population. In this article, we have considered how early detection of invasive pathogens can be aided by the introduction of sentinel plants, which are alternative hosts that display symptoms of infection more quickly than the main host species. We have explored the trade-off between faster detection of the pathogen using sentinel plants and the potential for sentinel plants to increase transmission, which is likely if the onset of symptoms is associated with high infectivity. Overall, our results indicate that sentinel plants have the potential to facilitate substantial reductions in the EDP for a wide range of sampling efforts (Figs 4A, 4B, 4C and 5B). The practical benefits of a reduced EDP are multiple. A lower prevalence of infection on pathogen discovery minimises the number of plants that must be removed, with benefits including lowering the total value of damaged crops, a lower reduction in crop yield and a reduced logistical cost of plant removal. Fast discovery of an invading pathogen also limits the opportunity for dispersal to other locations, meaning that incorporating sentinel plants into surveillance programmes may be beneficial for disease containment on a broader scale [14,26]. Discovering the pathogen at a low prevalence may increase the chance that local eradication is feasible, although evaluating whether the benefits of sentinel plants are sufficient to allow this would require control strategies to be accounted for explicitly in our model.

In addition to the overall potential of sentinel plants to facilitate early detection, our results highlight that both the optimal number of sentinel plants to introduce to the population and the optimal proportion of sentinel plants to include in each sample vary according to the sampling resources available (Figs 3, 4, 5A). The deployment of sentinel plants can be substantially less beneficial, or even detrimental, if either of these quantities are chosen without carefully assessing the impact of the monitoring programme in advance (Figs 3A, 4A, 4B, 4C).

In this study, our focus was investigating the degree to which including sentinel plants in a surveillance programme could reduce the EDP. Although our analyses suggest that sentinel plants may be effective for this purpose, there is clear motivation to consider these results within a wider economic context. In practice, an important consideration is the cost of implementing the surveillance programme, which must be compared against the benefits of fast detection of the invading pathogen. Further research is needed to quantify the economic costs of surveillance and control, accounting for the cost of sampling as well as the cost of removing infected plants and the resulting loss in plant value. The specific objective of the control strategy also needs to be carefully considered. For example, if reducing the opportunity for the pathogen to be exported to a new location prior to detection is a particular focus, the decision maker may wish to consider the ‘global’ epidemic cost associated with pathogen exportation in addition to the ‘local’ cost incurred in the controlled region [20,26], or to seek surveillance strategies that minimise the detection prevalence in the sentinel population as well as the crop population (for a preliminary analysis, see S4 Text and S14 Fig).

Although here we considered *X*. *fastidiosa* infection in *O*. *europaea* and *C*. *roseus* as a case study, our model provides a general framework that can be used to assess sentinel-based surveillance strategies in other scenarios. For example, we could consider alternative sentinel species by adjusting the model parameters appropriately. One possibility for an alternative sentinel species is alfalfa, a known host of many *X*. *fastidiosa* strains. A benefit of alfalfa is that it is itself economically valuable, which may increase its attractiveness to growers. However, alfalfa has a longer presymptomatic period than *C*. *roseus*, of around 6–9 months [67], which could limit its effectiveness as a sentinel host. An exploration of this trade-off may require the incorporation of economic considerations in our epidemiological modelling approach. Our framework could similarly be adapted to apply to alternative pathosystems. For example, monitoring programmes for citrus greening disease (a bacterial infection of citrus with causal agents *Candidatus* Liberibacter spp.) are hindered by a long period of presymptomatic infection in mature trees [37,38]. Younger trees develop symptoms more quickly [37,42], but also tend to support more rapid growth rates in the vector population [68,69]; our model could be used to explore their suitability for use as sentinel plants, taking this drawback into account.

An important limitation of our modelling framework is that we did not include vector dynamics explicitly. Since our goal was to use the simplest possible model to investigate the potential of sentinel plants to facilitate early detection of invasive pathogens, we instead assumed that vector behaviour was captured implicitly through the model parameterisation (specifically, through the parameters *β*_*C*_ and *β*_*S*_, which characterise the rate at which the pathogen is transmitted between hosts). Whilst a similarly implicit characterisation of vector behaviour has been used in previous modelling studies (see, for example, [26,27,61]), it does limit the possibility of altering the assumptions underlying the vector dynamics and observing the resulting effects on epidemiological dynamics. For example, our model assumes that vectors are equally attracted to crop and sentinel plants. If instead vectors exhibit a strong preference for crop plants, or are simply more likely to feed on them due to their much larger size, the relative rate at which sentinel plants become infected may be too low for them to be useful as early warning beacons for infection. On the other hand, if vectors preferentially feed on sentinel plants their benefit may be greater, as the rate at which crop plants become infected will be reduced. Similarly, we assume that vectors make no distinction between hosts that are displaying symptoms and hosts that are not. This is not necessarily the case–for example, vectors of *X*. *fastidiosa* in grapevine appear to prefer feeding on nonsymptomatic hosts rather than symptomatic ones [70]. Such a preference may alter the transmission dynamics between crop and sentinel plants, since sentinels are assumed to remain nonsymptomatic for a shorter period of time than crop plants. These dynamics can be approximated in our model by altering the transmission rate parameters (see S15 Fig for an example in which the susceptibility to infection of sentinels is half that of crop plants). However, extending our model to include explicit vector compartments would allow us to perform a thorough investigation into the influence of vector ecological dynamics on the effectiveness of sentinel-based surveillance strategies.

Throughout, we assumed that visual inspection was perfectly sensitive for identifying infection in Detectable plants and not at all sensitive for Undetectable plants, with no distinction between crop and sentinel hosts (see Section 2.3). However, allowing for false negatives in Detectable plants and/or positives in Undetectable plants would affect the EDP and the optimal number of sentinels to include in the population for a given sample size and sample interval. If the chance of testing errors differed between crop and sentinel hosts, the optimal allocation of sampling effort between crops and sentinels would vary. Although a detailed exploration of the impact of varying detection sensitivities is beyond the scope of this study, this is a key focus of our ongoing research.

Our model could also be extended to incorporate additional realism in other ways. For example, we could extend the transmission model considered here to account for the spatial structure of the population, allowing the likelihood of transmission between any two plants to depend on the distance between them [19,21,26–28,30]. Considering a spatially heterogeneous model would raise additional questions regarding the optimal spatial placement of sentinel plants and their selection as part of a monitoring programme, particularly if the risk of pathogen invasion also varied in space [71]. In addition to spatial heterogeneities, we could also consider temporal heterogeneities in the probability of invasion and detection that arise due to seasonal effects that impact vector dynamics [55,72] and the level of symptoms displayed by infected hosts [36,73,74]. These heterogeneities motivate considering temporally varying sampling strategies that allow sampling resources to be optimally allocated throughout the year. Further avenues for investigation include incorporating a latent period (time from when infection occurs to when the plant becomes infectious) in the model, or more generally a graduated progression through model compartments in which symptom expression and/or the likelihood of detection increase between successive compartments [75–77].

In summary, our results represent a step towards understanding how sentinel plants may be used to facilitate the early detection of invasive plant pathogens. As we have shown, sentinels have the potential to reduce the expected incidence of disease upon pathogen discovery substantially, thereby increasing the chance of pathogen containment and lowering the cost of reactive control. These results encourage further research into the economic and logistical viability of using sentinel plants as a means of combatting the problem of presymptomatic or asymptomatic infection, as well as into the precise epidemiological characteristics of particular sentinel-crop-pathogen combinations. Monitoring programmes involving sentinel plants have the potential to reduce the negative impacts of a range of invading plant pathogens.

## Supporting information

S1 Text Random sampling vs. repeated sampling. (PDF)

S2 Text Variation in parameter values. (PDF)

S3 Text Bayesian optimisation. (PDF)

S4 Text Variation in the objective function. (PDF)

S1 Table Parameter variation in the baseline case: the parameters that we varied, their meanings, their values used in the main text and the alternative values we considered in our Supplementary analyses. (PDF)

S2 Table Parameter variation with sentinels included: the parameters that we varied, their meanings, their values used in the main text and the alternative values we considered in our Supplementary analyses. (PDF)

S1 Fig Resultant EDPs (as a percentage of the total population) for the example system described in S1 Text, illustrating how random sampling outperforms repeated sampling. (PDF)

S2 Fig The effect of varying the model parameters on the baseline EDP. (PDF)

S3 Fig The effect of varying the model parameters on the baseline EDP. (PDF)

S4 Fig The effect of varying the transmission coefficient for ‘Detectable’ sentinels from *β*_*S*_ = 5×10^−5^ (baseline value) to *β*_*S*_ = 2.5×10^−5^ (A,C,E) and to *β*_*S*_ = 1×10^−4^ (B,D,F). (PDF)

S5 Fig The effect of varying the transmission scaling factor for ‘Undetectable’ crops from *ε*_*C*_ = 0.015 (baseline value) to *ε*_*C*_ = 0.1 (A,C,E) and to *ε*_*C*_ = 0.25 (B,D,F). (PDF)

S6 Fig The effect of varying the transmission scaling factor for ‘Undetectable’ sentinels from *ε*_*S*_ = 0.1 (baseline value) to *ε*_*S*_ = 0.02 (A,C,E) and to *ε*_*S*_ = 0.5 (B,D,F). (PDF)

S7 Fig The effect of varying the mean duration of the crop ‘Undetectable’ period from *γ*_*C*_ = 452 days (baseline value) to *γ*_*C*_ = 350 days (A,C,E) and to *γ*_*C*_ = 550 days (B,D,F). (PDF)

S8 Fig The effect of varying the mean duration of the sentinel ‘Undetectable’ period from *γ*_*S*_ = 49 days (baseline value) to *γ*_*S*_ = 28 days (A,C,E) and to *γ*_*S*_ = 70 days (B,D,F). (PDF)

S9 Fig The effect of varying the crop population size from *P*_*C*_ = 1000 plants (baseline value) to *P*_*C*_ = 500 plants (A,C,E) and to *P*_*C*_ = 1500 plants (B,D,F). (PDF)

S10 Fig The effect of varying the initial number of infected plants from *U*_0_ = 1 (baseline value) to *U*_0_ = 2 (A,C,E) and to *U*_0_ = 4 (B,D,F). (PDF)

S11 Fig Schematic summarising how the EDP was obtained for an example sampling strategy. (PDF)

S12 Fig Resultant EDPs in the baseline case and for the optimal strategies shown in Figs 3 and 4 of the main text. (PDF)

S13 Fig The optimal proportion of available sentinels to include in the sample as the sample size (*N*) and sample interval (Δ) vary, for the system outlined in Section 3.3 and Fig 5 of the main text. (PDF)

S14 Fig Optimising the number of sentinels to include in the population and sample when Ω = EDP+(0.5×EDP_sent_). (PDF)

S15 Fig Optimising the number of sentinels to include in the population and sample when the susceptibility of sentinels to infection is halved. (PDF)
