# Supplementary material for: Using ‘sentinel’ plants to improve early detection of invasive plant pathogens
Source: PLoS Comput Biol. 2023 Feb 2;19(2):e1010884. doi: 10.1371/journal.pcbi.1010884 (PMC9928126; doi:10.1371/journal.pcbi.1010884)
Supplement: S1 Text — (PDF) [file pcbi.1010884.s001.pdf]

**Using 'sentinel' plants to improve early detection of invasive plant pathogens**

Francesca A. Lovell-Read, Stephen Parnell, Nik J. Cunniffe, Robin N. Thompson

## S1 Text. Random sampling vs. repeated sampling

When considering the use of sentinel plants for surveillance, as well as deciding how many sentinels to add to the population ($P_S$), an important question is how many of those sentinels to include in the sample ($N_S$). A natural choice is to sample the maximum possible number of sentinels – that is, choosing $N_S = \min(P_S, N)$. However, in some circumstances this would result in frequent resampling of the same plants. For example, if the total sample size $N$ is equal to the total number of sentinels in the population $P_S$, selecting $N_S$ in this way would mean inspecting an identical set of plants on every sampling round. Since the information we gain about the disease status of a plant we have already inspected in the recent past is less than the information we gain by looking at a plant we have not previously inspected, repeated sampling of this kind may reduce the effectiveness of the surveillance strategy compared to a situation in which the sample selection has an element of randomness.

Here, we use a simple example to demonstrate how repeated sampling of the same plants can lead to a worse outcome than selecting plants to sample at random. We constructed a standard one-species Susceptible-Infected (SI) model in which plants progress from the 'Susceptible' class to the 'Infected' class at rate $\beta SI$ (where $\beta$ is the transmission coefficient, $S$ is the number of 'Susceptible' plants and $I$ is the number of 'Infected' plants). Setting $\beta = 5 \times 10^{-6}$, we generated simulated epidemic curves in a population of size $1000$, beginning from a single infected individual. We considered a monitoring strategy in which samples of size $N$ were taken from the population every $\Delta$ days, with detection occurring if and only if an 'Infected' plant was included in the sample. We implemented this monitoring strategy on our simulated epidemic curves for a range of sample sizes $N$ and sample intervals $\Delta$. For each choice of $N$ and $\Delta$ we considered both random sampling (in which the sampled plants were chosen at random on every sampling round), and repeated sampling (in which the same $N$ plants were inspected on every sampling round).

For the chosen model parameterisation, random sampling outperformed repeated sampling across the range of $(N, \Delta)$ values considered (S1 Fig). This result is conceptually equivalent to the phenomenon observed in the main text – that sampling as many sentinels as possible is not always the best strategy, if repeated sampling is required to achieve this. Note that the

difference between the two sampling approaches was smaller for large sample intervals $\Delta$, since the longer the time between samples the less relevant the information gained from a previous sample is to the current state of the system. Therefore, less information is lost by looking at the same plants. The difference between the two approaches also decreased for larger samples sizes $N$, since detection occurred within fewer sampling rounds (thus less repetition occurred).
