# Supplementary material for: Using ‘sentinel’ plants to improve early detection of invasive plant pathogens
Source: PLoS Comput Biol. 2023 Feb 2;19(2):e1010884. doi: 10.1371/journal.pcbi.1010884 (PMC9928126; doi:10.1371/journal.pcbi.1010884)
Supplement: S2 Text — (PDF) [file pcbi.1010884.s002.pdf]

Using ‘sentinel’ plants to improve early detection of invasive plant pathogens

Francesca A. Lovell-Read, Stephen Parnell, Nik J. Cunniffe, Robin N. Thompson

S2 Text. Variation in parameter values

Where possible, the epidemiological parameters used in our main analyses were chosen based on literature estimates for *X. fastidiosa* infection in *O. europaea* and *C. roseus* (Table 1 of the main text). However, due to uncertainty in these reported values and the lack of available estimates of some parameters, we also conducted supplementary analyses to investigate the robustness of our results to parameter variation. We performed these analyses both for the baseline case and with sentinels included. The parameters that we varied, their meanings, their values taken in the main text and the alternative values considered in our supplementary analyses are given in S1 Table (baseline case) and S2 Table (with sentinels included). In almost all cases considered, the selected parameter was varied whilst all other parameters remained fixed at their main text values. The exception to this was when varying the crop population size (P_C): in those cases, we simultaneously scaled the transmission coefficients for ‘Detectable’ crops and sentinels (β_C and β_S respectively) to ensure that the epidemic growth rate remained fixed at $\beta_C P_C = 0.05$ [1]. In the baseline case, for each alternative parameter value considered we present plots analogous to Fig 2C in the main text, showing the baseline EDP as a percentage of the total crop population size (S2, S3 Figs). When sentinels were included, for each alternative parameter value considered we present plots analogous to Fig 5 in the main text, showing the optimal total number of sentinels in the population, the percentage change in EDP compared to the baseline level, and the resultant EDP (S4-S10 Figs).

References

1. White SM, Navas-Cortés JA, Bullock JM, Boscia D, Chapman DS. Estimating the epidemiology of emerging Xylella fastidiosa outbreaks in olives. Plant Pathol. 2020;69(8):1403-13. doi: 10.1111/ppa.13238.
