# Supplementary material for: Using ‘sentinel’ plants to improve early detection of invasive plant pathogens
Source: PLoS Comput Biol. 2023 Feb 2;19(2):e1010884. doi: 10.1371/journal.pcbi.1010884 (PMC9928126; doi:10.1371/journal.pcbi.1010884)
Supplement: S3 Text — (PDF) [file pcbi.1010884.s003.pdf]

Using 'sentinel' plants to improve early detection of invasive plant pathogens

Francesca A. Lovell-Read, Stephen Parnell, Nik J. Cunniffe, Robin N. Thompson

S3 Text. Bayesian optimisation

Overview of Bayesian optimisation

Bayesian optimisation is an efficient technique for finding the extrema of objective functions that are expensive to evaluate [1]. It is an iterative process in which successive (possibly noisy) observations of an objective function are taken at carefully selected trial points and used to update our beliefs about its likeliest form. Beginning from a prior distribution on the objective function (invariably a Gaussian process prior [1, 2]), on each iteration the objective function is sampled at a trial point and Bayes' theorem is applied to incorporate the observed result into a posterior distribution. The extrema of this posterior approximate those of the objective function with increasing accuracy on each iteration.

Successive trial points are selected by maximising an acquisition function that quantifies the potential gain of sampling at a particular point. When seeking to minimise the objective function, the acquisition function is typically defined so that it takes high values i) near to previously sampled points where the objective function is small (known as exploitation), and ii) where there is a large amount of uncertainty, and the objective function has the potential to be small (known as exploration). In contrast to the objective function, the acquisition function is cheap to evaluate and maximise, so the best trial point can be found easily.

The judicious selection of trial points based on their utility makes Bayesian optimisation a particularly efficient method in terms of the number of evaluations of the objective function required, which is very useful when sampling is expensive [1]. Furthermore, Bayesian optimisation can be applied to an objective function that does not have a known analytic form, as long as the function can be evaluated computationally for a given choice of input parameters. It is therefore an ideal optimisation technique for this study, since our objective function is both analytically inaccessible and expensive to evaluate computationally.

Application of Bayesian optimisation in this study

In this study, we implemented Bayesian optimisation in MATLAB using the inbuilt function '*bayesopt*' [3, 4]. Since '*bayesopt*' seeks to minimise (rather than maximise) a specified objective function, we defined our objective to be the percentage change in EDP compared to

the baseline level for a given (N, Δ) pair. Therefore, smaller (more negative) values of the objective corresponded to greater reductions in the EDP.

Initially, we fixed the number of sentinels added to the population (P_S) and considered the number of sentinels included in the sample (N_S) as the single optimisable variable, allowed to vary between 0 and the (constant) upper bound $\min(P_S, N)$ (see Section 3.2 of the main text). We used the '*expected-improvement-plus*' acquisition function, which selects the next trial point (in this case value of N_S) so as to maximise the expected reduction in the objective function compared to the current estimated minimum, whilst also avoiding overexploitation of any given area [3]. For each choice of (N, Δ) we performed 30 iterations of the Bayesian optimisation algorithm (30 iterations is the '*bayesopt'* default); that is, we evaluated the objective function for 30 successively selected values of N_S (not necessarily distinct). Each evaluation of the objective function involved computing the resultant EDP for the sampling strategy under consideration by performing sampling on 25,000 simulated epidemic curves (as shown in S11 Fig), and comparing it to the baseline EDP for the specified N and Δ.

Subsequently, we allowed both the number of sentinels added to the population (P_S) and the number of sentinels included in the sample (N_S) to vary simultaneously, and adapted the Bayesian optimisation algorithm to treat each of these as an optimisable variable (see Section 3.3 of the main text). We allowed P_S to vary between 0 and 350, and N_S to vary between 0 and $\min(P_S, N)$ (now variable). Since the default implementation of '*bayesopt*' requires constant bounds for the optimisable variables, we first specified that N_S could vary between 0 and the total sample size N. Then, we further restricted the feasible region from which trial points could be drawn by enforcing the constraint $N_S \leq P_S$. This form of constraint (a deterministic function of the optimisable variables) can be implemented in MATLAB using the '*XConstraintFcn*' option in '*bayesopt*' [5].

Applying Bayesian optimisation in this study resulted in a substantial reduction in the number of objective function evaluations required, and therefore a much shorter computation time. For example, in Fig 5 we considered 900 distinct (N, Δ) pairs. For each of these pairs, P_S was allowed to vary in the range $[0,350]$, and N_S in the range $\min(P_S, N)$. Seeking the optimal combination of P_S and N_S by exhaustive search would have required tens of thousands of

objective function evaluations for each (N, Δ) pair; by applying Bayesian optimisation, this was reduced to 30 iterations per pair.

References

[1] Brochu, E., Cora, V.M. & de Freitas, N. 2010. A Tutorial on Bayesian Optimization of Expensive Cost Functions, with Application to Active User Modeling and Hierarchical Reinforcement Learning. *arXiv:1012.2599*. Available at: https://arxiv.org/pdf/1012.2599v1.pdf.

[2] Frazier, P.I. 2018. A Tutorial on Bayesian Optimization. *arXiv:1807.02811*. Available at: https://arxiv.org/abs/1807.02811.

[3] MathWorks. 2021. *Bayesian Optimization Algorithm*. (Accessed 8th March 2022). Available at: https://uk.mathworks.com/help/stats/bayesian-optimization-algorithm.html

[4] MathWorks. 2021. *bayesopt*. (Accessed 8th March 2022). Available at: https://uk.mathworks.com/help/stats/bayesopt.html

[5] MathWorks. 2021. *Constraints in Bayesian Optimization*. (Accessed 8th March 2022). Available at: https://uk.mathworks.com/help/stats/constraints-in-bayesian-optimization.html
