# Supplementary material for: Using ‘sentinel’ plants to improve early detection of invasive plant pathogens
Source: PLoS Comput Biol. 2023 Feb 2;19(2):e1010884. doi: 10.1371/journal.pcbi.1010884 (PMC9928126; doi:10.1371/journal.pcbi.1010884)
Supplement: S4 Text — (PDF) [file pcbi.1010884.s004.pdf]

Using ‘sentinel’ plants to improve early detection of invasive plant pathogens

Francesca A. Lovell-Read, Stephen Parnell, Nik J. Cunniffe, Robin N. Thompson

S4 Text. Variation in the objective function

Throughout our main analyses, we investigated how including sentinel plants in a surveillance strategy could reduce the EDP compared to the baseline level, where the EDP was defined to be the expected detection prevalence in the crop population at the time of discovery. In doing so, we assumed that minimising the prevalence of infection amongst valuable crop plants was likely to be the primary objective of the grower, and did not consider the prevalence of infection in the sentinel population. However, in practice a grower may also wish to take the expected detection prevalence in the sentinel population ($\mathrm{EDP}_{\mathrm{sent}}$) into account in some way when evaluating the effects of surveillance strategies. Reasons for this include reducing the cost of removing infected plants post-detection and reducing the likelihood that the pathogen is exported to a new location before control can be implemented. Additionally, in some pathosystems the sentinel plants may have some intrinsic value – for example, young orange trees used as sentinels for greening disease in citrus groves (see Discussion).

Our analyses can be adapted to explore any balance of interest between crop and sentinel discovery prevalence by replacing the EDP with $\Omega = \mathrm{EDP} + (\omega_S \times \mathrm{EDP}_{\mathrm{sent}})$ for some weight $\omega_S \in [0,1]$. Computationally speaking, this simply requires a small adjustment to the objective function used in our Bayesian optimisation algorithm. Our main analyses correspond to the case $\omega_S = 0$ (Fig 5): here, we display analogous results for the case $\omega_S = 0.5$ (S14 Fig). This represents a hypothetical situation in which an infected sentinel plant is considered half as concerning as an infected crop plant (rather than not being considered at all). In this case, introducing a large number of sentinel plants is penalised by contributing to an increased $\mathrm{EDP}_{\mathrm{sent}}$. The optimal number of sentinels to include in the population is therefore reduced compared to the case in which $\omega_S = 0$, apart from when the sample interval Δ is large and the optimal number of sentinels is equal to the sample size (i.e. not in excess) (S14A Fig). The achievable percentage reduction in Ω compared to the baseline level is also reduced compared to the case in which we consider solely the EDP, since infected sentinel plants are no longer discounted (S14B Fig). This confirms that the specific objective a grower wishes to achieve should be carefully considered before the results of sentinel-based surveillance strategies are evaluated.
