# Supplementary material for: Using ‘sentinel’ plants to improve early detection of invasive plant pathogens
Source: PLoS Comput Biol. 2023 Feb 2;19(2):e1010884. doi: 10.1371/journal.pcbi.1010884 (PMC9928126; doi:10.1371/journal.pcbi.1010884)
Supplement: S2 Table — (PDF) [file pcbi.1010884.s006.pdf]

Using 'sentinel' plants to improve early detection of invasive plant pathogens

Francesca A. Lovell-Read, Stephen Parnell, Nik J. Cunniffe, Robin N. Thompson

S2 Table.

Table S2. Parameter variation with sentinels included: the parameters that we varied, their meanings, their values used in the main text and the alternative values we considered in our Supplementary analyses.

Parameter	Meaning	Main text value	Alternative values considered
β_S	Transmission coefficient for 'Detectable' sentinels	5×10^{-5}	$2.5 \times 10^{-5}, 1 \times 10^{-4}$
ε_C	Transmission scaling factor for 'Undetectable' crops	0.015	$0.1, 0.25$
ε_S	Transmission scaling factor for 'Undetectable' sentinels	0.1	$0.02, 0.5$
γ_C	Mean duration of crop 'Undetectable' period	452 days	350 days, 550 days
γ_S	Mean duration of sentinel 'Undetectable' period	49 days	28 days, 70 days
P_C	Number of crop plants in the population	1000	$500, 1500$*
U_0	Initial number of 'Undetectable' infected individuals	1	$2, 4$

* values of β_C, β_S scaled accordingly to ensure $\beta_C P_C = 0.05$.
