# Supplementary material for: Using ‘sentinel’ plants to improve early detection of invasive plant pathogens
Source: PLoS Comput Biol. 2023 Feb 2;19(2):e1010884. doi: 10.1371/journal.pcbi.1010884 (PMC9928126; doi:10.1371/journal.pcbi.1010884)
Supplement: S1 Fig — (PDF) [file pcbi.1010884.s007.pdf]

Using 'sentinel' plants to improve early detection of invasive plant pathogens

Francesca A. Lovell-Read, Stephen Parnell, Nik J. Cunniffe, Robin N. Thompson

S1 Fig.

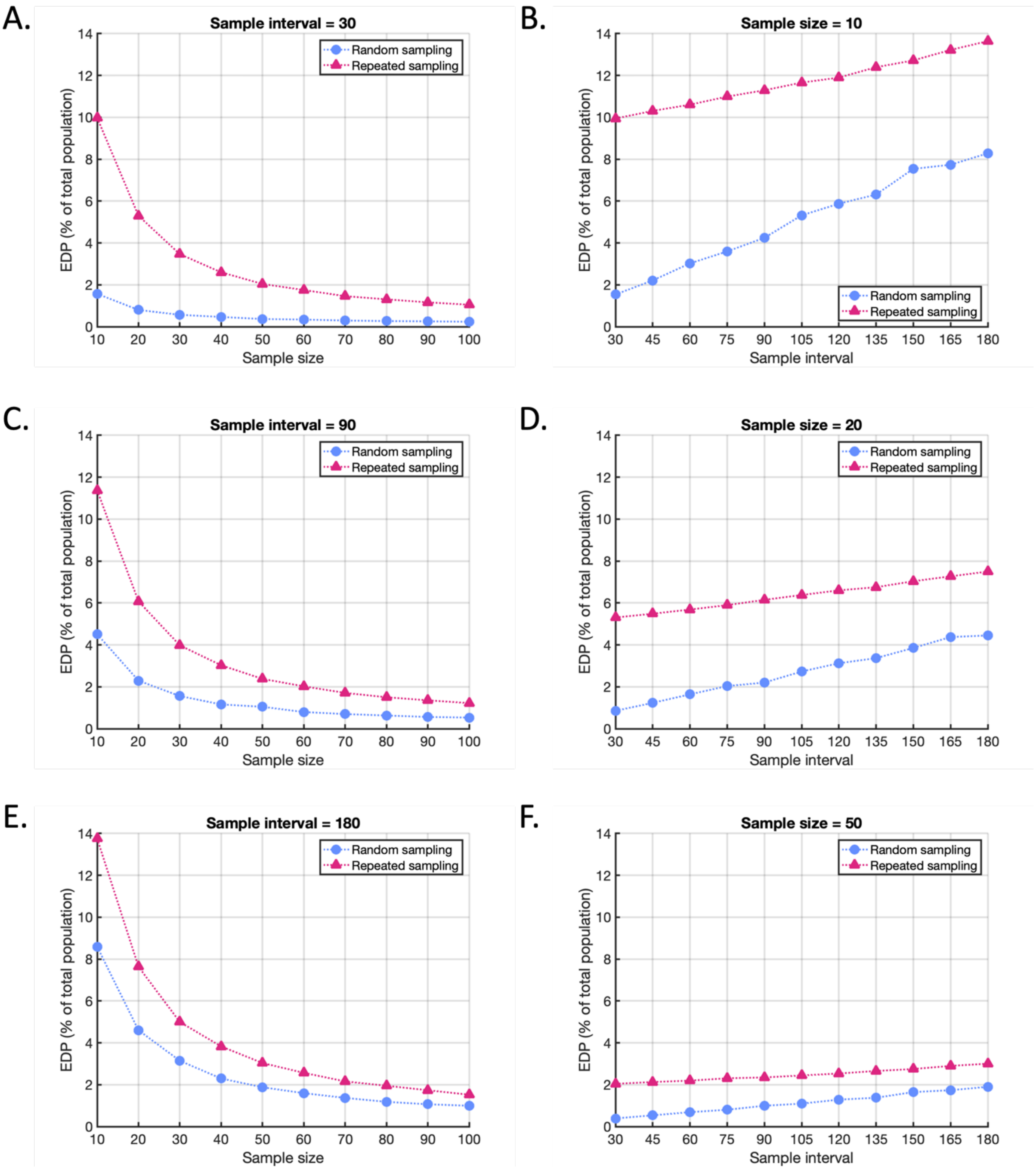

S1 Fig. Resultant EDPs (as a percentage of the total population) for the example system described in S1 Text, illustrating how random sampling (blue circles) outperforms repeated sampling (pink triangles). A,C,E. The resultant EDP for sample intervals $\Delta = 30$ days (A), 90 days (C) and 180 days (E) as the sample size N varies. B,D,F. The resultant EDP for sample sizes $N = 10$ (B), $N = 20$ (D) and $N = 50$ (F) as the sample interval varies.
