# Supplementary material for: Using ‘sentinel’ plants to improve early detection of invasive plant pathogens
Source: PLoS Comput Biol. 2023 Feb 2;19(2):e1010884. doi: 10.1371/journal.pcbi.1010884 (PMC9928126; doi:10.1371/journal.pcbi.1010884)
Supplement: S2 Fig — (PDF) [file pcbi.1010884.s008.pdf]

Using 'sentinel' plants to improve early detection of invasive plant pathogens

Francesca A. Lovell-Read, Stephen Parnell, Nik J. Cunniffe, Robin N. Thompson

S2 Fig.

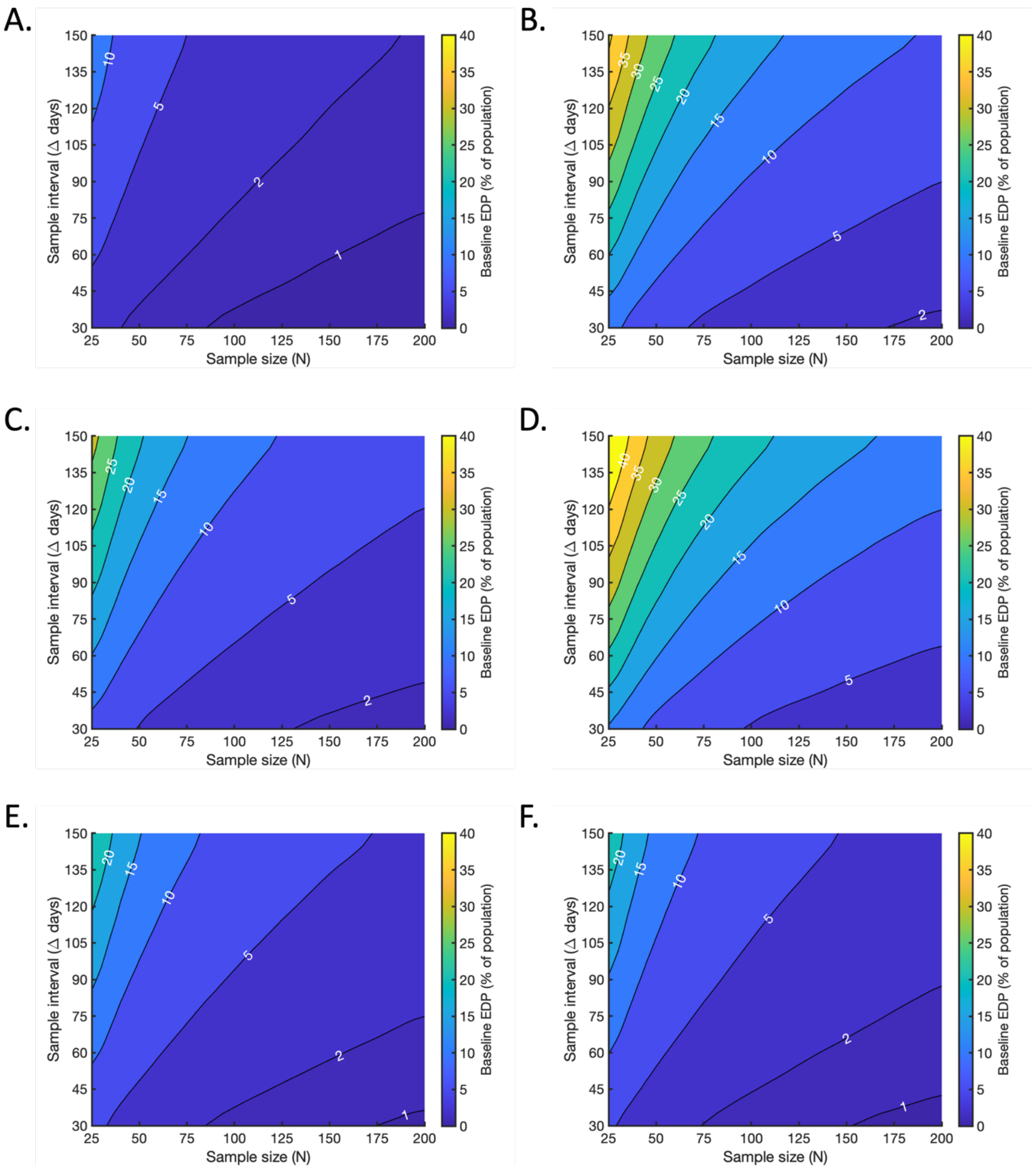

S2 Fig. The effect of varying the model parameters on the baseline EDP. Panels analogous to Fig 2C in the main text. A,B. The effect of varying the transmission coefficient for 'Detectable' crops (β_C) from its baseline value ($\beta_C = 5 \times 10^{-5}$) to $\beta_C = 2.5 \times 10^{-5}$ (A) and to $\beta_C = 1 \times 10^{-4}$ (B). C,D. The effect of varying the transmission scaling factor for 'Undetectable' crops (ε_C) from its baseline value ($\varepsilon_C = 0.015$) to $\varepsilon_C = 0.1$ (C) and to $\varepsilon_C = 0.25$ (D). E,F. The effect of varying the mean duration of the crop 'Undetectable' period (γ_C) from its baseline value (452 days) to $\gamma_C = 350$ days (E) and to $\gamma_C = 550$ days (F).
