# Supplementary material for: Using ‘sentinel’ plants to improve early detection of invasive plant pathogens
Source: PLoS Comput Biol. 2023 Feb 2;19(2):e1010884. doi: 10.1371/journal.pcbi.1010884 (PMC9928126; doi:10.1371/journal.pcbi.1010884)
Supplement: S3 Fig — (PDF) [file pcbi.1010884.s009.pdf]

Using ‘sentinel’ plants to improve early detection of invasive plant pathogens

Francesca A. Lovell-Read, Stephen Parnell, Nik J. Cunniffe, Robin N. Thompson

S3 Fig.

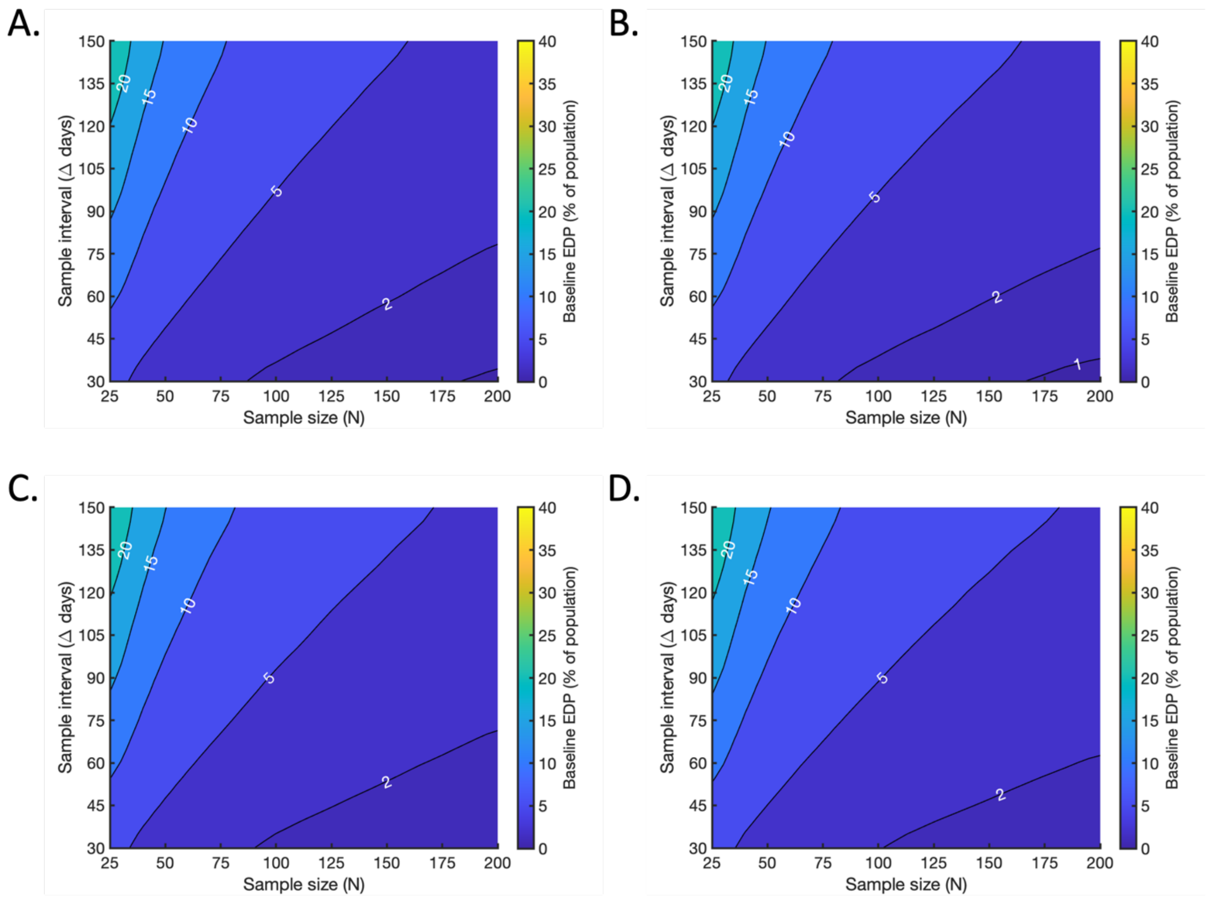

S3 Fig. The effect of varying the model parameters on the baseline EDP. Panels analogous to Fig 2C in the main text. A,B. The effect of varying the crop population size (P_C) from its baseline value (1000) to $P_C = 500$ (A) and to $P_C = 1500$ (B). C,D. The effect of varying the initial number of 'Undetectable' infected hosts (U_0) from its baseline value ($U_0 = 1$) to $U_0 = 2$ (C) and to $U_0 = 4$ (D).
