# Supplementary material for: Using ‘sentinel’ plants to improve early detection of invasive plant pathogens
Source: PLoS Comput Biol. 2023 Feb 2;19(2):e1010884. doi: 10.1371/journal.pcbi.1010884 (PMC9928126; doi:10.1371/journal.pcbi.1010884)
Supplement: S5 Fig — (PDF) [file pcbi.1010884.s011.pdf]

**Using ‘sentinel’ plants to improve early detection of invasive plant pathogens**

Francesca A. Lovell-Read, Stephen Parnell, Nik J. Cunniffe, Robin N. Thompson

# S5 Fig.

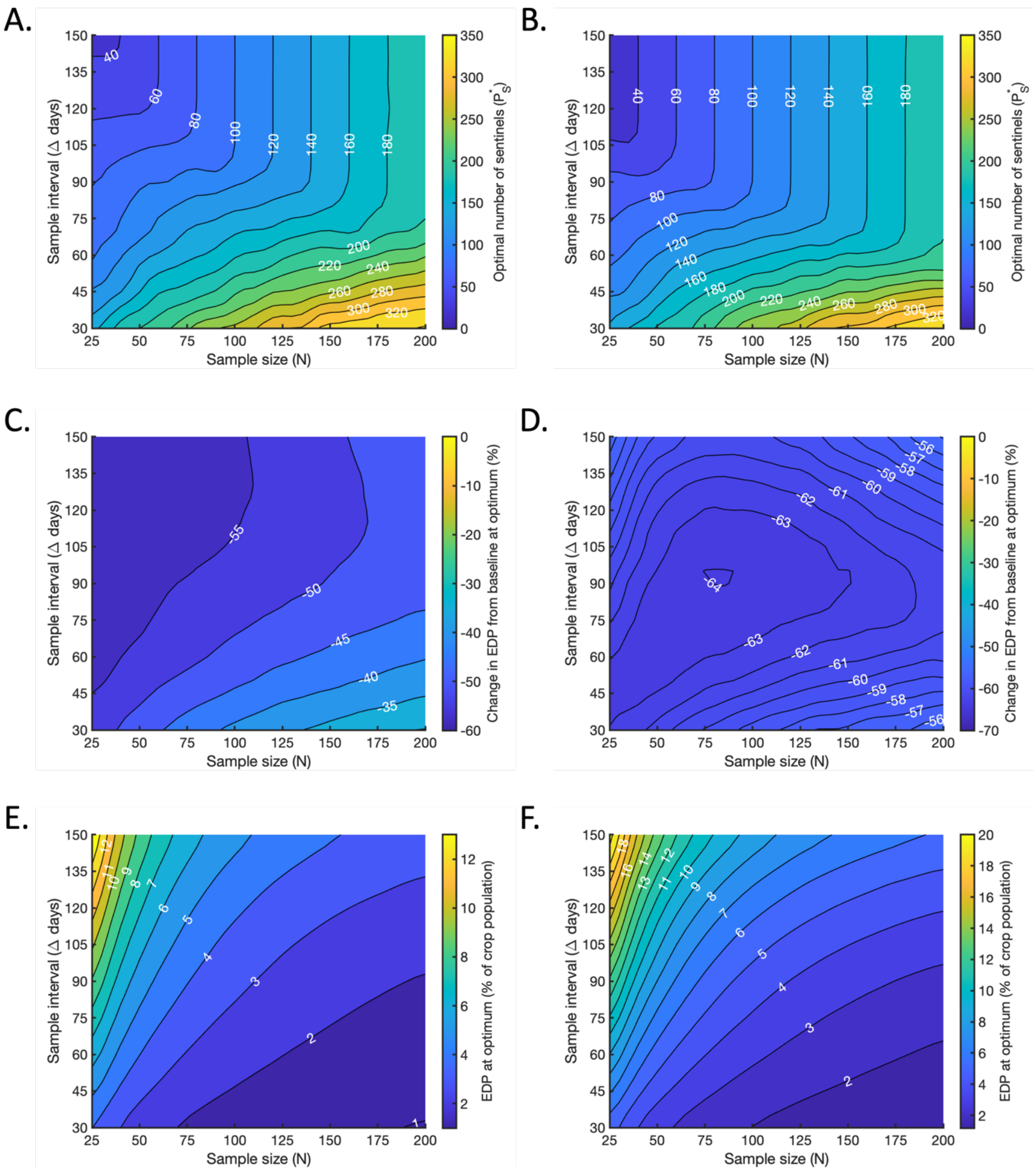


**S5 Fig. The effect of varying the transmission scaling factor for ‘Undetectable’ crops from $\varepsilon_C = 0.015$ (baseline value) to $\varepsilon_C = 0.1$ (A,C,E) and to $\varepsilon_C = 0.25$ (B,D,F). Panels analogous to Fig 5 in the main text.** A,B. The optimal number $P_S^*$ of sentinel plants to include in the population, for which the maximal reduction in the EDP compared to the baseline level is achieved (if $N_S$ is also chosen optimally). C,D. The percentage change in the EDP compared to the baseline value at the optimum, achieved when $P_S = P_S^*$ and $N_S = N_S^*$. E,F. The resultant value of the EDP at the optimum, expressed as a percentage of the total crop population.
