# Supplementary material for: Using ‘sentinel’ plants to improve early detection of invasive plant pathogens
Source: PLoS Comput Biol. 2023 Feb 2;19(2):e1010884. doi: 10.1371/journal.pcbi.1010884 (PMC9928126; doi:10.1371/journal.pcbi.1010884)
Supplement: S6 Fig — (PDF) [file pcbi.1010884.s012.pdf]

Using ‘sentinel’ plants to improve early detection of invasive plant pathogens

Francesca A. Lovell-Read, Stephen Parnell, Nik J. Cunniffe, Robin N. Thompson

S6 Fig.

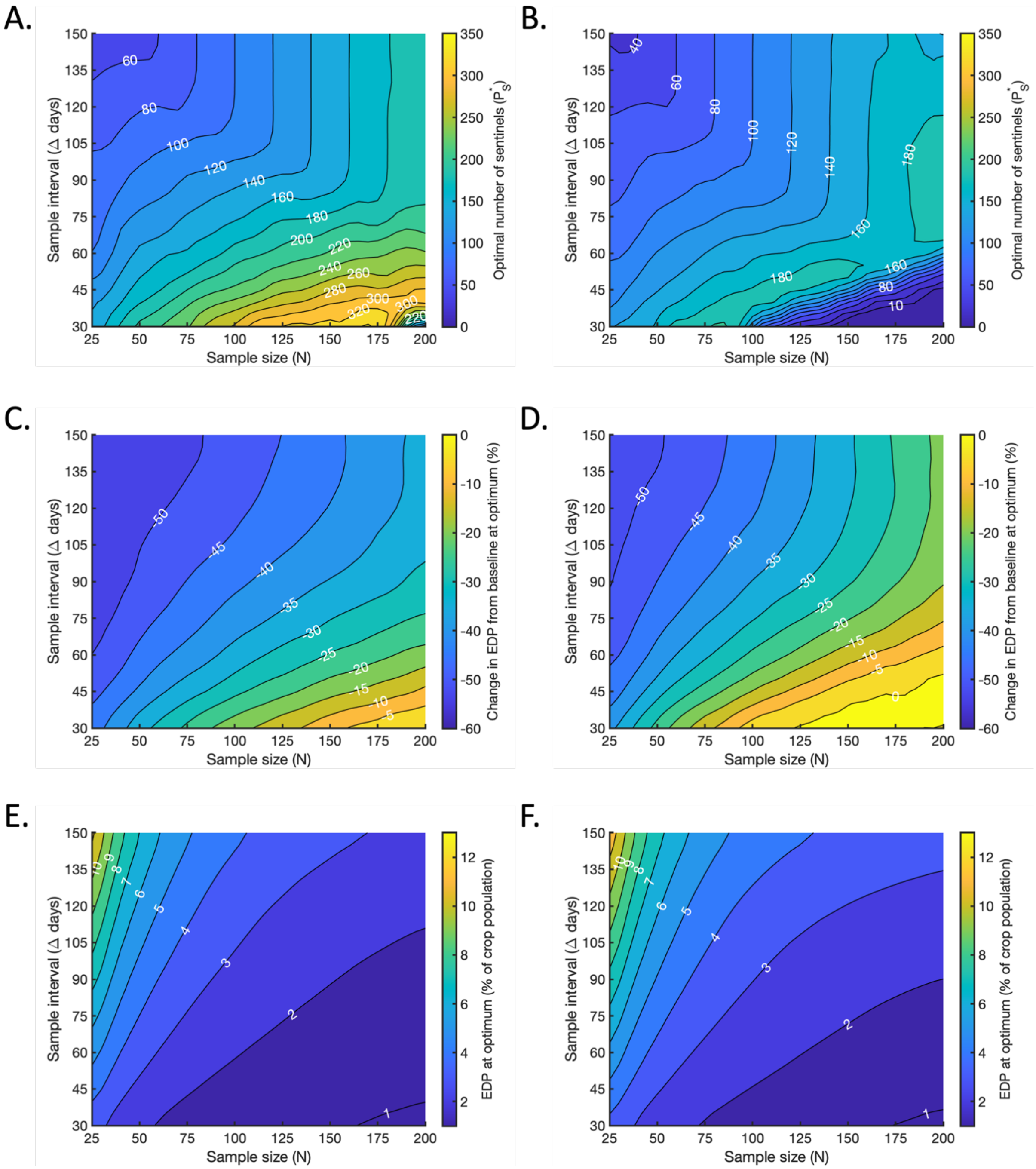

S6 Fig. The effect of varying the transmission scaling factor for ‘Undetectable’ sentinels from $\varepsilon_S = 0.1$ (baseline value) to $\varepsilon_S = 0.02$ (A,C,E) and to $\varepsilon_S = 0.5$ (B,D,F). Panels analogous to Fig 5 in the main text. A,B. The optimal number P_S^* of sentinel plants to include in the population, for which the maximal reduction in the EDP compared to the baseline level is achieved (if N_S is also chosen optimally). C,D. The percentage change in the EDP compared to the baseline value at the optimum, achieved when $P_S = P_S^*$ and $N_S = N_S^*$. E,F. The resultant value of the EDP at the optimum, expressed as a percentage of the total crop population.
