# Supplementary material for: Using ‘sentinel’ plants to improve early detection of invasive plant pathogens
Source: PLoS Comput Biol. 2023 Feb 2;19(2):e1010884. doi: 10.1371/journal.pcbi.1010884 (PMC9928126; doi:10.1371/journal.pcbi.1010884)
Supplement: S11 Fig — (PDF) [file pcbi.1010884.s017.pdf]

Using 'sentinel' plants to improve early detection of invasive plant pathogens

Francesca A. Lovell-Read, Stephen Parnell, Nik J. Cunniffe, Robin N. Thompson

S11 Fig.

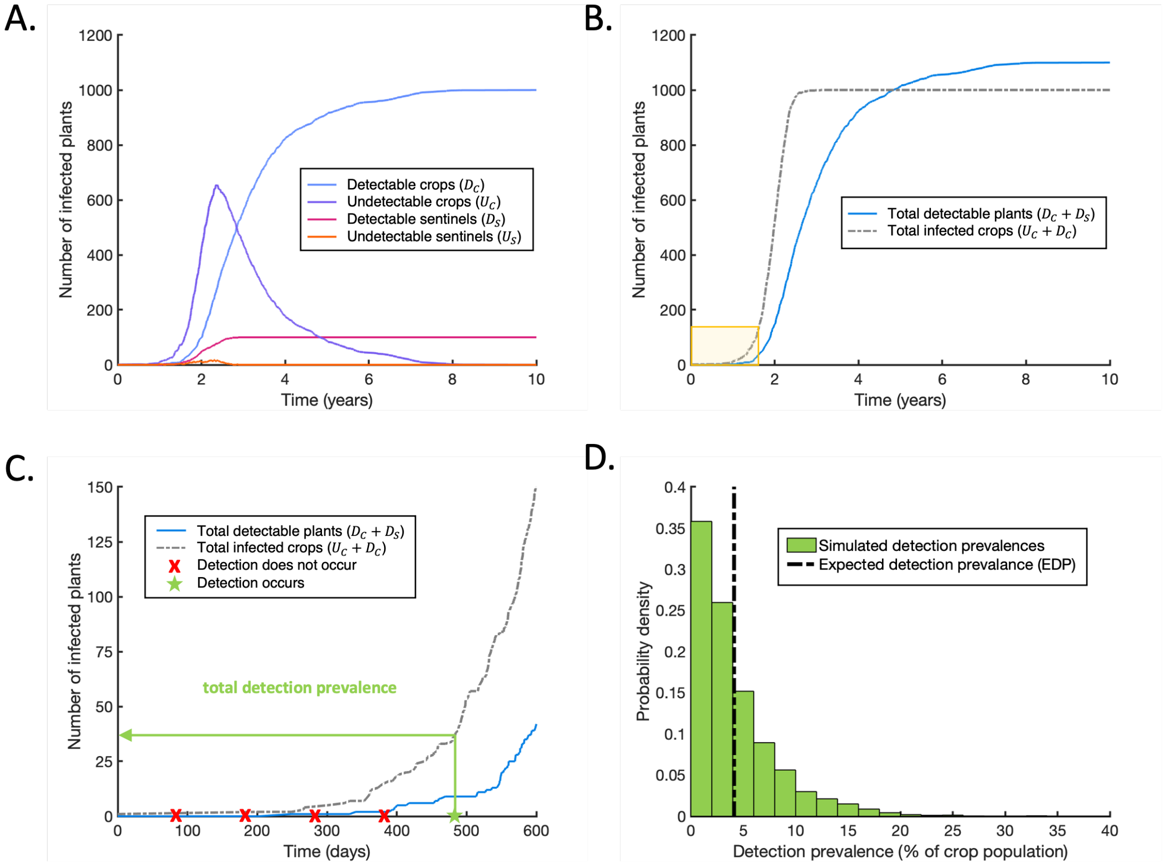

S11 Fig. Schematic summarising how the EDP was obtained for an example sampling strategy. A. Stochastic simulations of pathogen spread in the model system were performed using the Gillespie SSA (direct method). In each simulation the number of 'Undetectable' and 'Detectable' crops and sentinels were tracked over time until the entire population became infected. In the simulation shown, we considered a population of $P_C = 1000$ crops and $P_S = 100$ sentinels, beginning with a single 'Undetectable' individual. All epidemiological parameters were as specified in Table 1 of the main text. B. The total number of 'Detectable' plants in the population ($D_C + D_S$; blue solid line) and the total number of infected crop plants in the population ($U_C + D_C$; grey dash-dotted line). Yellow box highlights the region that is shown magnified in panel C. C. A magnification of the yellow region highlighted in panel B, showing how the sampling strategy (defined in terms of the parameters N, N_C, N_S and Δ) is implemented on an individual epidemic curve. Random samples of size $N = N_C + N_S$ were taken from the population every Δ days, with detection occurring as soon as a 'Detectable' plant (D_C or D_S) was present in the sample. In this example, we used $N_C = 50, N_S = 50, \Delta = 100$ days. The time of the first sample was selected uniformly at random from the interval [0,100]. When detection occurred, the total detection prevalence amongst crop plants ($U_C + D_C$) was recorded. D. The sampling strategy described in C was repeated on 10000 simulated epidemic curves. Due to the inherent randomness of the spread and sampling simulations, different values for the total

detection prevalence were obtained each time, leading to a probability distribution for this prevalence (green bars). The EDP was calculated as the mean of this distribution (black dash-dotted line).
