# Supplementary material for: Using ‘sentinel’ plants to improve early detection of invasive plant pathogens
Source: PLoS Comput Biol. 2023 Feb 2;19(2):e1010884. doi: 10.1371/journal.pcbi.1010884 (PMC9928126; doi:10.1371/journal.pcbi.1010884)
Supplement: S12 Fig — (PDF) [file pcbi.1010884.s018.pdf]

Using 'sentinel' plants to improve early detection of invasive plant pathogens

Francesca A. Lovell-Read, Stephen Parnell, Nik J. Cunniffe, Robin N. Thompson

S12 Fig.

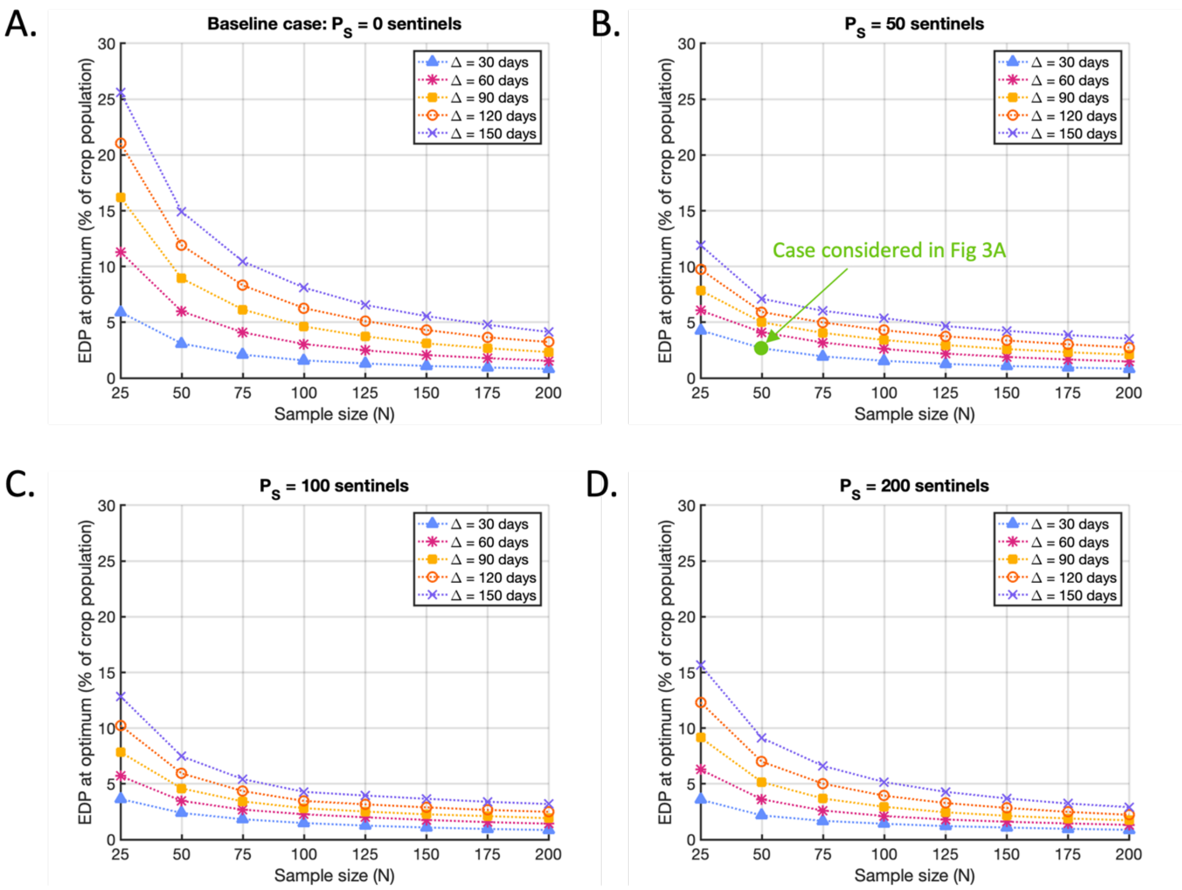

S12 Fig. Resultant EDPs in the baseline case and for the optimal strategies shown in Figs 3 and 4 of the main text. A. The EDP in the baseline case ($P_S = 0$), where sample size (N) and sample interval (Δ) vary as shown. Since the baseline EDP depends on N and Δ, relative changes in the EDP compared to this baseline for different values of N and Δ (Figs 4ABC of the main text) are not a measure of the resultant EDP. B. The best achievable resultant EDP when $P_S = 50$, corresponding to the optimal strategies identified in Fig 3B in the main text. Green circle marks the case considered in Fig 3A in the main text ($P_S = 50, N = 50, \Delta = 30$ days). C. The analogous figure to B, but with $P_S = 100$ sentinels added to the population and results corresponding to the strategies identified in Fig 3C. D. The analogous figure to B, but with $P_S = 200$ sentinels added to the population and results corresponding to the strategies identified in Fig 3D.
