# Supplementary material for: Using ‘sentinel’ plants to improve early detection of invasive plant pathogens
Source: PLoS Comput Biol. 2023 Feb 2;19(2):e1010884. doi: 10.1371/journal.pcbi.1010884 (PMC9928126; doi:10.1371/journal.pcbi.1010884)
Supplement: S13 Fig — (PDF) [file pcbi.1010884.s019.pdf]

Using 'sentinel' plants to improve early detection of invasive plant pathogens

Francesca A. Lovell-Read, Stephen Parnell, Nik J. Cunniffe, Robin N. Thompson

S13 Fig.

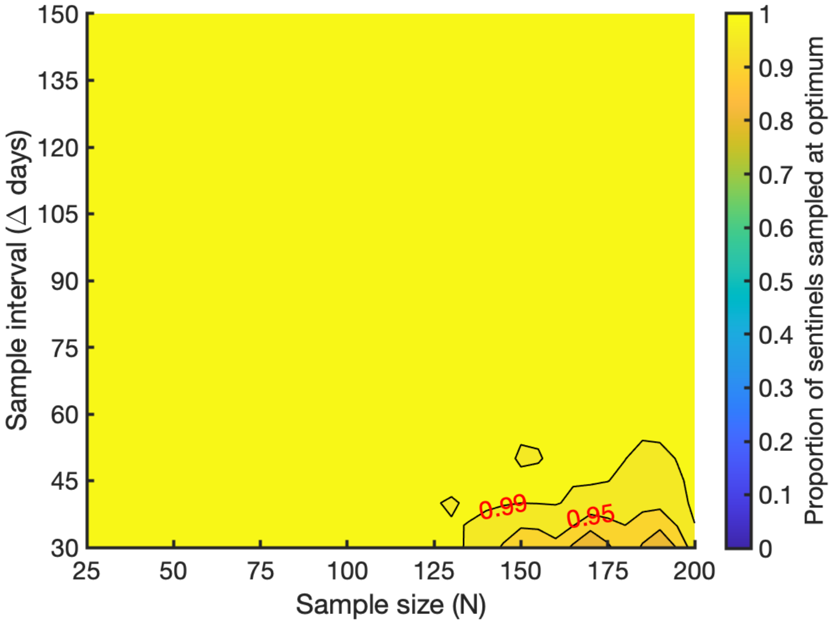

S13 Fig. The optimal proportion of available sentinels to include in the sample as the sample size (N) and sample interval (Δ) vary, for the system outlined in Section 3.3 and Fig 5 of the main text. For each (N, Δ) pair, this proportion is computed as the optimal number of sentinels to include in the sample (N_S^*) divided by the maximum possible number of sentinels that could be included ($\min(P_S^*, N)$), where N_S^*, P_S^* are obtained from the Bayesian optimisation algorithm.
