# Supplementary material for: Using ‘sentinel’ plants to improve early detection of invasive plant pathogens
Source: PLoS Comput Biol. 2023 Feb 2;19(2):e1010884. doi: 10.1371/journal.pcbi.1010884 (PMC9928126; doi:10.1371/journal.pcbi.1010884)
Supplement: S14 Fig — (PDF) [file pcbi.1010884.s020.pdf]

Using 'sentinel' plants to improve early detection of invasive plant pathogens

Francesca A. Lovell-Read, Stephen Parnell, Nik J. Cunniffe, Robin N. Thompson

S14 Fig.

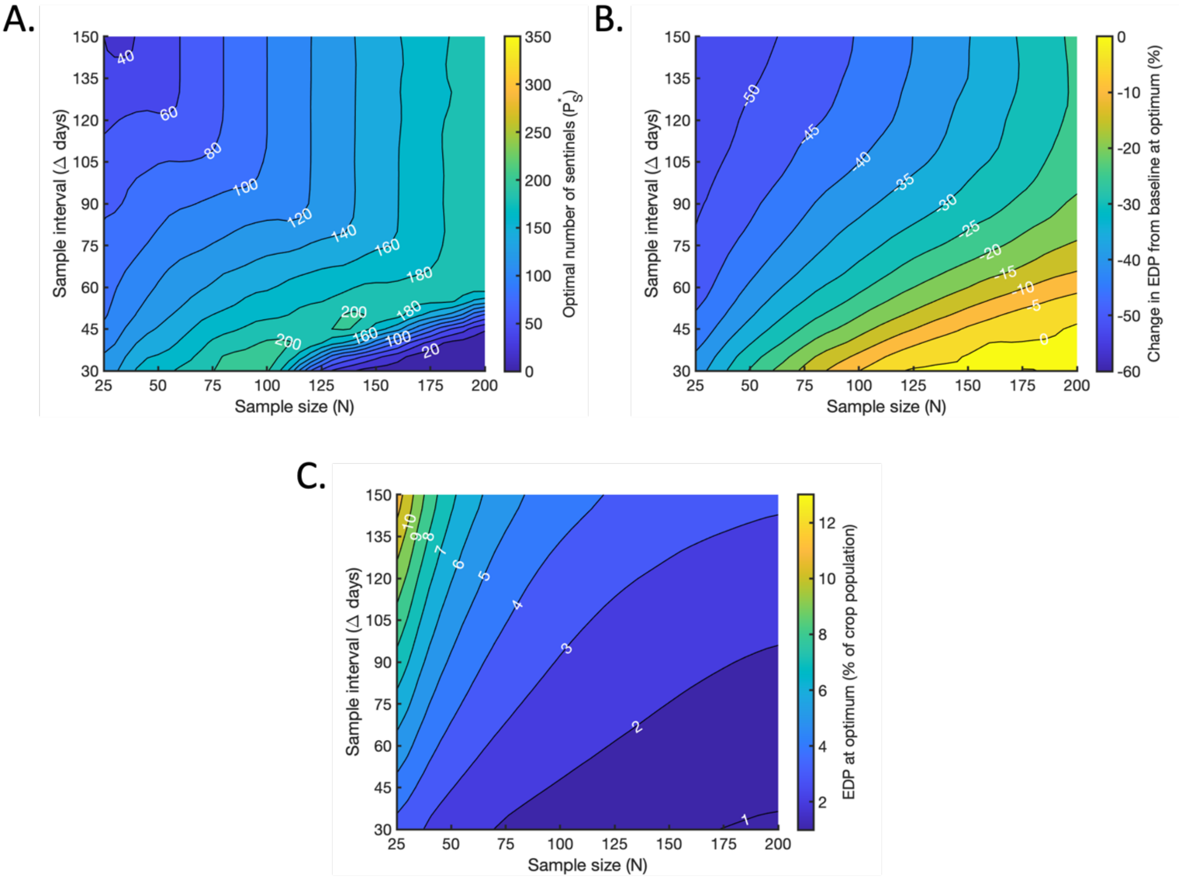

S14 Fig. Optimising the number of sentinels to include in the population and sample when $\boldsymbol{\Omega = \mathrm{EDP} + (0.5 \times \mathrm{EDP}_{\mathrm{sent}})}$. A. The optimal number P_S^* of sentinel plants to include in the population, for which the maximal reduction in Ω compared to the baseline level is achieved (if N_S is also chosen optimally). B. The percentage change in Ω compared to the baseline value at the optimum, achieved when $P_S = P_S^*$ and $N_S = N_S^*$. C. The resultant value of Ω at the optimum, expressed as a percentage of the total crop population.
