# Supplementary material for: Using ‘sentinel’ plants to improve early detection of invasive plant pathogens
Source: PLoS Comput Biol. 2023 Feb 2;19(2):e1010884. doi: 10.1371/journal.pcbi.1010884 (PMC9928126; doi:10.1371/journal.pcbi.1010884)
Supplement: S15 Fig — (PDF) [file pcbi.1010884.s021.pdf]

Using 'sentinel' plants to improve early detection of invasive plant pathogens

Francesca A. Lovell-Read, Stephen Parnell, Nik J. Cunniffe, Robin N. Thompson

S15 Fig.

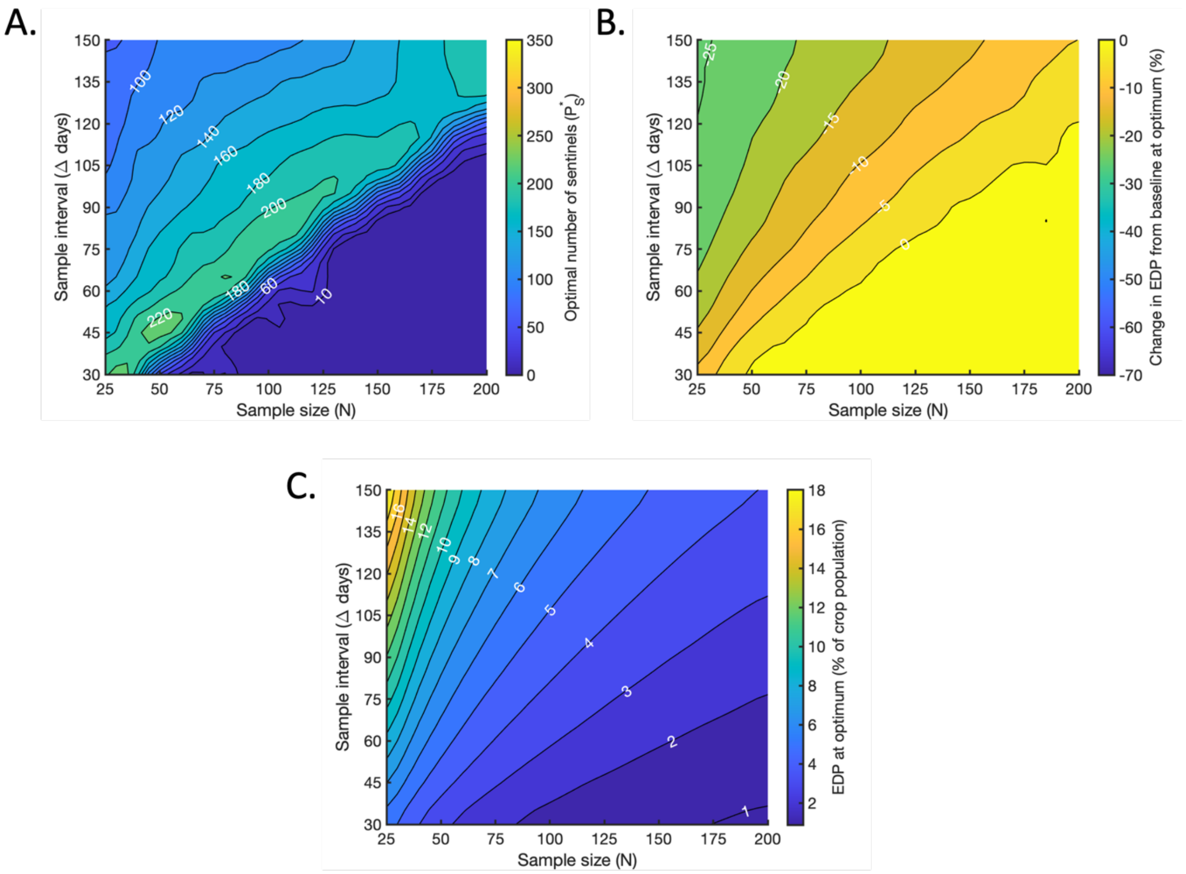

S15 Fig. Optimising the number of sentinels to include in the population and sample when the susceptibility of sentinels to infection is halved. A. The optimal number P_S^* of sentinel plants to include in the population, for which the maximal reduction in Ω compared to the baseline level is achieved (if N_S is also chosen optimally). B. The percentage change in Ω compared to the baseline value at the optimum, achieved when $P_S = P_S^*$ and $N_S = N_S^*$. C. The resultant value of Ω at the optimum, expressed as a percentage of the total crop population.
